# Impacts of Tourism on Wild Elephant Behavior in a Protected Area: Thresholds for Sustainable Wildlife Viewing

**DOI:** 10.1002/ece3.72842

**Published:** 2026-01-12

**Authors:** Brooke Friswold, Antoinette van de Water, Ave Owen, Megan English, Tommaso Savini, Liv Baker, Chution Savini, George Gale

**Affiliations:** ^1^ Conservation Ecology Program King Mongkut's University of Technology Thonburi Bangkok Thailand; ^2^ Bring the Elephant Home Prachuap Khiri Khan Thailand; ^3^ The School for Field Studies Phnom Penh Cambodia; ^4^ Sarah Lawrence College Bronxville New York USA; ^5^ International College for Sustainability Studies, Srinakharinwirot University Bangkok Thailand

**Keywords:** animal behavior, Asian elephants, ecotourism, ethology, human–wildlife interactions, Thailand, wildlife management

## Abstract

Wildlife tourism can support conservation but also imposes stress on wildlife, particularly cognitively complex and social species like Asian elephants ( *Elephas maximus* ), highlighting the need for science‐based management regulations. We assessed the behavioral responses of wild Asian elephants in Kuiburi National Park, Thailand, against varying levels of tourism pressure using scan and focal sampling over three years, including during park closures. We hypothesized that tourism pressure influences elephant behavioral responses, with the strength and nature of responses varying according to disturbance intensity, demographics, and environmental variables. Tourism pressure was measured at two scales: sighting tourism pressure (STP; number of people, vehicles, distance, and noise at each elephant sighting) and Daily Tourism Pressure (DTP; total daily tourists). Elevated numbers of vehicles, people, noise, and close distances significantly increased rates of stress‐related, vigilance, and passive aggressive behaviors while reducing affiliative behavior and prompting retreat. The most consistently selected tourism‐related predictors of behavior were number of vehicles, number of people, proximity to humans, and noise level, with affiliative behavior emerging as the most sensitive indicator of disturbance. Behavioral thresholds were identified for “ideal” and “acceptable” tourism conditions: > 100 m/125 m viewing distance, < 4/< 8 vehicles, < 10/< 21 people, and noise < 32/< 42 dB, beyond which negative behavioral responses increased significantly. Following park re‐openings, elephant detections declined, especially for cow‐calf groups, indicating increased avoidance and vulnerability to tourism of socially cohesive groups. These results support evidence‐based regulations for wildlife tourism, including the use of thresholds for management such as buffer zones, quiet viewing protocols, daily and sighting vehicle and visitor caps, guide training, and adaptive seasonal closures. Integrating empirically derived behavioral thresholds into protected area and national policies provides a scalable and transferable model to reduce disturbance, improve animal welfare, and promote ethical and sustainable elephant tourism in Thailand and beyond.

## Graphical abstract

Wildlife tourism can benefit conservation but also causes stress in socially and cognitively complex species like Asian elephants. Using multi‐year behavioral observations in Kuiburi National Park, Thailand, we identified clear thresholds for tourism variables (e.g., vehicles, people, distance, noise) beyond which elephants exhibited increased stress and aggression, reduced affiliative behavior, and increased spatial avoidance. These findings provide actionable guidelines for regulating tourism to enhance elephant welfare and promote sustainable viewing practices.

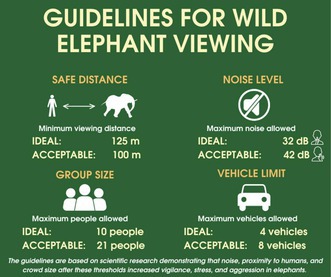

## 1 Introduction

Wildlife tourism presents a multifaceted challenge. Its revenue plays a significant role in safeguarding habitats and wildlife (Reynolds and Braithwaite 2001; Higginbottom 2004) through establishing protected areas, supporting conservation programs (Stronza et al. 2019), increasing economic development for local communities, and the promotion of pro‐environmental attitudes (Tablado and D'Amico 2017). However, poorly managed tourism can have unintended consequences (Li et al. 2017) such as increasing human‐wildlife conflict (Bagheriyan et al. 2023), habitat degradation (Ahmad et al. 2019), and altering animal behavior (Ngoprasert et al. 2007). These can lead to diminished wildlife health, welfare, conservation efforts, and local community livelihoods (Cui and Xu 2021). This paradox highlights a key challenge in conservation; although tourism can support conservation (Bookbinder et al. 1998), tourist presence and behavior can create stressors that undermine these very efforts (Maréchal et al. 2011; Ranaweerage et al. 2015). Increased tourist presence has been associated with elevated stress levels and behavioral modifications in wildlife (Piñeiro et al. 2012; Szott, Pretorius, and Koyama 2019). While some impacts are obvious, such as disease and collisions (Green and Giese 2004; Rendall et al. 2021), less perceptible disruptions to animal behavior and physiology can have equally significant consequences for fitness and welfare (Tyagi et al. 2019; Szott 2020). To ensure that wildlife tourism is not having a negative impact on fitness and welfare, research must assess how tourist pressure influences behavioral responses in wildlife to inform management practices (Moorhouse et al. 2015). There is growing support for ethical wildlife ecotourism practices that ensure welfare (Thomsen et al. 2023) and move from commodified experiences to a value‐based model (Belicia and Islam 2018) that evaluates and incorporates tractability, socio‐economic values, conservation, welfare, and ecosystem health (Meyer et al. 2021).

Elephants are among the most desired animals for wildlife tourism (Naidoo et al. 2016; Mangachena and Pickering 2021) and provide significant emotional value to human experiences (Curtin 2009; Van de Water et al. 2022). However, their high cognitive abilities and complex social structures (Byrne and Bates 2007; Bates et al. 2008) make them particularly susceptible to negative psychological impacts from tourism (Bradshaw et al. 2005; Plotnik and Jacobson 2022). Higher tourist pressure has been shown to increase stress levels and aggression in Asian elephants ( *Elephas maximus* ; Ranaweerage et al. 2015) and can also alter their movement patterns, activity budgets, and social interactions in African savanna elephants ( *Loxodonta africana* ; Szott, Pretorius, and Koyama 2019; Szott, Pretorius, Ganswindt, and Koyama 2019). These behavioral changes can have cascading physiological impacts that affect health, reproductive success, and survival (Tang et al. 2020). In severe cases, chronically stressed individuals may abandon key habitats (Patterson et al. 2025). Despite these concerns, wild elephant tourism often supports broader conservation efforts by protecting habitats and supporting biodiversity (Clubb and Mason 2002) with wild elephant tourism providing higher welfare conditions than captive alternatives (Veasey 2006; Bradshaw and Schore 2007; Clubb et al. 2008; Cohen 2009; Jacobs et al. 2022). Tourist preferences are shifting towards wild elephant tourism (Flower, Burns, and Jones 2021) with many tourists considering elephant welfare when selecting tourism activities (Flower, Burns, Jones, and McBroom 2021)—underscoring the need for management strategies that protect elephant welfare and support sustainable and ethical tourism options (Moorhouse et al. 2015).

Elephant tourism is a major source of income for many countries, including Thailand, and offers ecological (Harich et al. 2016), cultural, and economic value (Naidoo et al. 2016; Hayward et al. 2022; Van de Water et al. 2023). In addition to captive venues, Thailand offers opportunities to observe wild elephants in protected areas (Bansiddhi et al. 2019), where they are legally protected under laws such as the Prevention of Animal Cruelty and Provision Animal Welfare Act (Kingdom of Thailand 2014). However, there is no national legislation regulating wild elephant tourism, and while some parks enforce local measures, Thailand does not currently have evidence‐based nationwide guidelines. Ethological research (studying wildlife responses to human activity) can inform such protocols, supporting elephant well‐being, visitor safety, and broader conservation goals (Curio 1996; Parr et al. 2008; Buckley et al. 2016; Chock et al. 2025).

Kuiburi National Park (KNP) is one of two protected areas in Thailand offering safari‐style wild elephant tourism—supporting local livelihoods and conservation (Parr et al. 2008). The COVID‐19 pandemic caused tourism disruptions (Sumanapala and Wolf 2022), prompting park closures mandated by the Department of National Parks (DNP), which continued seasonally. These closures, along with fluctuating visitation levels, provided a unique opportunity to systematically assess elephant behavioral responses to varying tourism pressure. Such studies can reveal thresholds of behavioral change (Anderson et al. 2023; Moorhouse et al. 2015), with behavior studies often detecting impacts before downstream consequences emerge (Chock et al. 2025). Animal behavior studies also emphasize a non‐intrusive research approach (Baker et al. 2024) to improve ecotourism sustainability and guide evidence‐based tourism management (Millspaugh et al. 2007; Tang et al. 2020). Though some impacts of tourism on Asian elephants have been documented (Ranaweerage et al. 2015; Grant et al. 2023), further research and regulations are needed to ensure ethical and sustainable practices based on scientifically identified thresholds (Fennell 2022; Chock et al. 2025).

To address these gaps in knowledge, we examined how tourism pressure influences wild elephant behavior in Kui Buri National Park (KNP) across different levels of visitation intensity and time periods. Specifically, we asked: (i) How do varying levels of tourism pressure affect behavioral expressions in wild elephants and how do demographic and environmental factors modulate these responses? (ii) At what thresholds of disturbance variables (distance to humans, noise levels, and the number of vehicles and people) do abrupt behavioral shifts occur? (iii) How do behavioral responses and detection rates vary temporally across open and closed tourist seasons, particularly following re‐opening?

We hypothesized that tourism pressure influences the behavioral responses of wild elephants in a variety of contexts. To evaluate this overarching hypothesis, we developed the following specific predictions:

(P1) Behavioral expression: Elevated tourism pressure will increase vigilance, stress‐related, and aggressive behaviors while reducing affiliative behaviors and altering activity budgets (time spent in specific behaviors and states). The strength and direction of behavioral responses will vary with tourism‐related variables (distance to the closest human, noise level, and the number of people and vehicles), elephant demographics, and environmental context.
(P2) Tourism‐related thresholds: Behavioral variation will be most strongly associated with tourism‐related factors. Threshold responses to tourism‐related variables are expected, where abrupt behavioral shifts occur beyond specific levels of disturbance. Tourism‐related variables alter elephant movement patterns relative to humans.
(P3) Temporal dynamics: Elephant behavioral responses and detection rates will differ between periods of high and low tourism activity, including open and closed seasons, and will vary by elephant demographics, particularly around park opening and closing transitions

## 2 Methodology

### 2.1 Study Site

**FIGURE 1:**
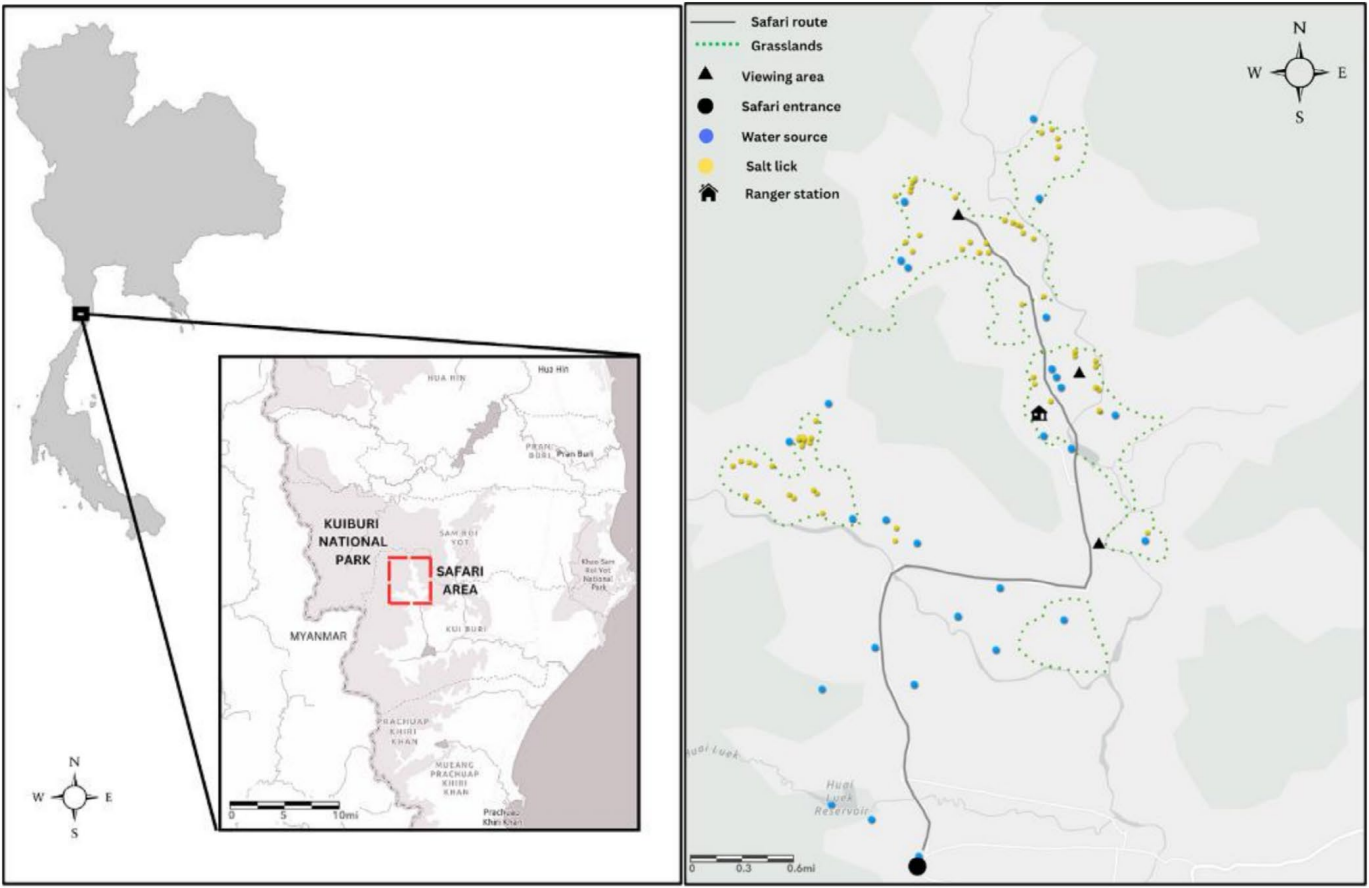
Left: Map of Kuiburi National Park, Thailand. Safari area with wild elephant tourism outlined in red. Right: 30 km^2^ safari route in Kuiburi National Park showing areas of interest along the route.

Kuiburi National Park is located in Prachuap Khiri Khan province, southwestern Thailand, in the Tenasserim Mountain Range bordering Myanmar (Figure 1). The park spans 969 km^2^ and forms part of the Kaeng Krachan Forest Complex. It experiences a seasonal climate, with a rainy season from late April to early November and a dry season from December to early April (with local and yearly variation) with an annual rainfall of 857 mm and a mean annual temperature of 28°C (Srikrachang et al. 2009). The park's forest composition features dry and moist evergreen forests (~70%), deciduous forests (~30%), and regenerated former agricultural land using native and non‐native species (KNP, 1999; ~3%). KNP supports a population of wild Asian elephants, estimated at 237–350 individuals (Department of National Parks, Wildlife, and Plant Conservation [DNP] 2021), which comprises a substantial portion of Thailand's remaining wild elephant population (~4013–4422; Sukmasuang et al. 2024).

The park was developed through a collaborative effort involving the DNP, WWF Thailand, local communities, and the Public‐Private Partnership Offering for Wildlife and Ecosystem Resilience (Prasit and Waiapa 2018). A wildlife safari area (Figure 1) was established in 1999 by the Kui Buri Wildlife Ecotourism Club to promote community participation and income generation for local residents (Parr et al. 2008). This designated 30 km^2^ safari route traverses reforested former pineapple farms and includes four primary viewing areas in the Huay Luek Elephant Watching Area, including an elevated viewing tower and the Payang Ranger Station (Figure 1). Safari vehicles operate daily from 14:00 to 18:00 and legitimately claim a high probability of elephant sightings year‐round, with the open landscape facilitating easy observation. KNP implemented park closures during the study period from September 1–October 31 in 2022 and June 1–June 30 in 2023. In 2024, KNP was the only national park in Thailand not to implement a yearly closure period. During the course of the study, there were no explicit or enforced regulations regarding exact distances, noise levels, or number of tourists/vehicles. Some signage existed at the entry to the park encouraging safe distances and reduced noise, and park rangers were often present to ensure safety.

### 2.2 Data Collection

Data collection periods varied annually due to changes in park closure dates but were conducted primarily during the rainy season; 2022: October 1–November 30 (closure during October); 2023: June 1–August 15 (closure during June); 2024: October 1–December 15 (no closure period; Table 1). Observations were standardized to be conducted only along the safari route (Figure 1) and only during safari operation hours (14:00 to 18:00) 5 days/week (Tuesday–Saturday). Survey days were occasionally canceled/amended due to weather or park access disruptions. The safari route was driven systematically in a research vehicle while communicating with field guides and rangers to determine areas of elephant presence. In order to capture the greatest amount of behavioral data, any reported or encountered elephant(s) were sampled where possible. The primary investigator collected the majority of the data, accompanied by research assistants. When conducting surveys during the closed tourist season, one or more rangers also accompanied. KNP supplied data on the daily number of visitors and vehicles each year which were divided into quartiles to quantify tourism pressure (Table 2).

**TABLE 1:** Summary of survey effort during open and closed tourist seasons across three years (2022–2024).

Open tourist season					Closed tourist season			
Year	Days	Avg. focal samples/day	Focal sample total	Scan sample total	Days	Avg. focal samples/day	Focal sample total	Scan sample total
2022	20	3.53	70	34	15	6.31	72	37
2023	29	6.22	124	65	20	5.14	84	56
2024	29	4.12	189	89	—	—	—	—
Total	78	4.62	383	188	35	5.73	156	93

*Note:* Survey effort was consistent across years during open seasons, with sampling during closed seasons limited to 2022 and 2023 as no closed season occurred in 2024.

**TABLE 2:** (a) *Sighting Tourism Pressure* (STP): Number of people and vehicles, including research personnel and park officials, present at each sighting. (b) *Daily Tourism Pressure*: Total number of people and vehicles entering the park per day, categorized into quartiles.

Daily Tourism Pressure (DTP)		
**Tourism Pressure**		**No. People**
None	—	1–15
Low	Q1	16–43
Medium	Q2	44–62
High	Q3	63–82
Extreme	Q4	83–221
**Tourism Pressure**		**No. Vehicles**
None	—	1–7
Low	Q1	8–13
Medium	Q2	14–18
High	Q3	19–23
Extreme	Q4	24–52

Sighting Tourism Pressure (STP)		
**Tourism Pressure**		**No. People**
Low	Q1	1–4
Medium	Q2	5–20
High	Q3	21–35
Extreme	Q4	36–125
**Tourism Pressure**		**No. Vehicles**
Low	Q1	0–1
Medium	Q2	2–3
High	Q3	4–5
Extreme	Q4	6–30

*Note:* Values reflect recorded tourism levels during the study period in KNP, ranging from lowest to highest daily entries. “None” refers to park closure periods when only rangers and researchers were present; STP does not include “None” as it includes these individuals in its assessment and therefore it is not possible for no pressure at a sighting to exist. Values reflect recorded tourism levels during the study period in KNP, ranging from lowest to highest daily entries.

For every elephant(s) encountered, we recorded the following for the elephant detection dataset: date, time, number of elephants, GPS location, and herd type: classified as Lone‐bull (LB), Bull‐group (BG), Cow‐calf group (CC), or Mixed‐group (MG, containing combinations of the above categories). We also documented vegetation type (shrub, waterhole, road, open forest, dense forest, or grassland) and measured distance from elephants to the nearest forest edge and human (measured using Sig Sauer KILO2500 6X22 laser rangefinder).

Upon approaching each elephant sighting or when elephant(s) joined a sighting, we conducted a five‐minute scan sample to capture initial movement responses to our presence (Bateson and Martin 2021). Scan samples were conducted if humans were already present at a sighting. Distances to elephants varied due to conditions but were kept at > 50 m as much as possible; occasionally, a combination of factors related to movements of the elephants, safari drivers, rangers, and/or tourists would cause distances to be < 50 m. We assessed elephant spatial responses via directional movement by comparing elephant(s) locations from the middle of the group (if more than one individual) at the start and end of the five‐minute period, categorizing movement as: Approach (A)—decreased distance to observer(s); Stationary (S)—no change in distance; Walk‐by (W)—continued in their original direction; Partial Retreat (PR)—moved away but remained visible; or Full Retreat (FR)—moved away to become out of sight.

Following each scan sample, we conducted 14‐min focal samples on pseudo‐randomly selected elephants (Altmann 1974) using the ZooMonitor application (Lincoln Park Zoo 2016; Wark et al. 2019). We recorded both all‐occurrence (count‐based) and continuous (duration‐based) behaviors simultaneously, targeting approximately five focal samples per day. Sampling was limited to ten individuals per sighting, with no elephant sampled more than once per day. Only sub‐adult (9–14 years) and adult (≥ 15 years) elephants were sampled to maintain behavioral consistency, and all observations were conducted from distances of 30–500 m.

Due to field constraints, individual elephants could not be identified. We collected comprehensive data for both focal and scan samples across several categories: demographic variables: sex (cow/bull), age class (sub‐adult 9–14 years, adult ≥ 15 years), herd type (LB, BG, CC, MG), temporal gland streaming (yes/no), body condition score (1–5; Wijeyamohan et al. 2015), musth status, number of elephants visible, and distance to forest edge (measured using Sig Sauer KILO2500 6X22 laser rangefinder). Environmental variables included: weather (sunny, overcast, rainy), wind presence (noted by researcher if present at start of sample), GPS location, and vegetation type. Tourism‐related variables collected at the start of each focal sample included: the number of people (all visible in the vicinity of the sighting), the number of vehicles (all visible in the vicinity of the sighting), type(s) of vehicle(s) present (ranger motorbike, safari vehicle, research vehicle), the distance from focal elephant to closest person (measured using Sig Sauer KILO2500 6X22 laser rangefinder), and the ambient noise level at the start of the data collection period (measured in decibels using the NIOSH Sound Level Meter app).

### 2.3 Behavioral Classification

To classify and categorize elephant behaviors for scan and focal sampling analyses, we developed and tested an ethogram (Data S1) in collaboration with the non‐profit organization Bring The Elephant Home, incorporating principles from existing elephant ethograms (Langbauer Jr 2000; McComb et al. 2014; ElephantVoices 2018; Poole and Granli 2021). The ethogram included continuous and all‐occurrence behaviors and was sorted into six behavior category states: “relaxed (continuous only),” “affiliative,” “stress‐related,” “vigilant,” “passive aggressive” and “active aggressive.” We focused on these behavior categories as they have been identified in previous elephant research as sensitive indicators of social cohesion, disturbance response, and overall welfare (Plotnik and De Waal 2014; Szott 2020). They are also readily observable during short‐term encounters with tourists. All‐occurrence behaviors were recorded as binary instances to capture their count and frequency (e.g., trunk curl, head shake). And continuous behaviors were recorded as durations of time in each activity with a corresponding behavior category (e.g., grazing, locomotion), recorded as a change in behavior after 3 s of its exhibition.

### 2.4 Data Pre‐Processing

Behaviors were analyzed across two separate tourism pressure gradients: *Sighting Tourism Pressure* (STP), the total number of people and vehicles at each elephant sighting, including both tourists, research personnel, and park officials (Table 2). *Daily Tourism Pressure* (DTP), the number of tourist visitors and tourist vehicles entering the park per day for tourism activities based on entry logs provided by KNP. The DTP category “None” denoted when the park was closed to tourism; the presence of rangers, researchers, or maintenance staff (typically ≤ 15 people and ≤ 7 vehicles) was possible, but these individuals were not engaged in tourism‐related activities and thus excluded from DTP.

An a priori sample size estimate was conducted based on the median estimated Asian elephant population in KNP (237–350 individuals; DNP, 2024). The analysis was conducted assuming a medium effect size (Cohen's *f* = 0.25), requiring 174 observations to achieve 80% power at a 5% significance level. All observations were standardized based on a consistent sampling effort of 4 h per day, 5 days per week during the survey period, across all years (2022–2024). All statistical analyses were conducted using R (R Core Team 2018). The durations of continuous behaviors were aggregated across all focal observation bouts conducted between 14:00 and 18:00 and averaged to construct an overall activity budget for this time window (Stafford et al. 2011). Data were then stratified by sex, age class, and other relevant variables that may influence behavioral expression.

### 2.5 Statistical Analyses

All analyses were conducted to evaluate the overarching hypothesis that tourism pressure influences elephant behavioral responses, organized according to the three predictions described previously using the most appropriate statistical approach for each behavioral response or predictor type (GLMMs for continuous behavioral outcomes, nonparametric tests for ordinal movement responses, and segmented regressions for threshold detection). All tests were evaluated at *α* = 0.05. Non‐parametric analyses were used where data did not meet normality assumptions. Behavioral response variables were standardized across sampling periods to ensure comparability. Each analysis was planned a priori and addressed a specific question. Bonferroni adjustments were applied to post hoc pairwise comparisons following ANOVA and Kruskal–Wallis tests to control Type I error within those analyses. Because each model addressed a different behavioral outcome or ecological question, the analyses represent separate inferential families. For the main hypothesis‐driven models (GLMMs, temporal comparisons, and threshold analyses), formal correction across all tests was not applied because the analyses were conceptually independent. Nonetheless, we interpret *p*‐values in conjunction with effect sizes, model fit, and biological relevance when evaluating significance.

#### 2.5.1 P1: Behavioral Expression

To test whether elevated tourism pressure increased stress‐related, vigilance, and aggressive behaviors while reducing affiliative behaviors, we used Poisson Generalized Linear Mixed Models (GLMMs) fitted separately for each behavioral category. Active aggressive behaviors were analyzed where possible but limited in analysis due to low detection in certain contexts. Random intercepts for individual elephants and random slopes for tourist season were included. Fixed effects included tourism‐related predictors (number of vehicles, number of people, noise level, distance to closest person), environmental variables (windy conditions, date), and demographic factors (sex, herd type, number of elephants). Separate models were fitted for affiliative, passive‐aggressive, stress‐related, vigilant, and active‐aggressive behaviors. Models used a log link function, and parameter estimates, standard errors, and *p*‐values were extracted for each predictor. The direction and magnitude of effects were interpreted using model coefficients. Additionally, a time‐sensitive covariate representing the 30‐day adjustment period following park re‐opening was included in GLMMs to capture short‐term changes in behavior following shifts in tourism presence. Model performance was then assessed using Akaike's Information Criterion (AIC), with ΔAIC values calculated relative to the best‐supported model for each behavioral response. Models with lower AIC values were considered to have stronger empirical support. For each predictor, we report the coefficient of determination (*R* ^2^), standardized regression coefficient (*β*), and significance level (*p*‐value). This AIC‐based comparison provided an objective measure of variable importance, allowing the identification of key disturbance factors for subsequent threshold analyses.

Prior to constructing the final Generalized Linear Mixed Models (GLMMs), we examined associations among all predictor variables and behavioral outcomes using Spearman's rank correlations with pairwise complete observations to assess potential multicollinearity. Predictor variables were then selected for inclusion in the GLMMs based on theoretical expectations and previous literature on elephant behavioral responses to human disturbance (Ranaweerage et al. 2015; Szott, Pretorius, Ganswindt, and Koyama 2019). Variables with high pairwise correlations (|*r*| > 0.7) were not included together in the same model to prevent collinearity. This approach ensured that all model predictors were ecologically meaningful and grounded in established hypotheses.

To examine whether the duration of behaviors varied across levels of tourism pressure, we analyzed continuous behavioral data derived from focal sampling. Behavioral durations (in seconds per observation) were grouped by Sighting Tourism Pressure (STP) and because duration data were non‐normally distributed, Kruskal–Wallis *H*‐tests were used to assess differences across STP levels for each behavioral category, including “alert” (vigilance) and “flee/retreat” behaviors. Where significant main effects were detected, pairwise Mann–Whitney U tests with Bonferroni correction were applied to identify specific contrasts between STP levels.

To assess how behavioral responses varied by herd type we conducted a series of one‐way ANOVA tests comparing mean behavioral rates among herd types. This approach was used to isolate the effects of social grouping on behavior independently of other predictors. When significant effects were detected, post hoc Tukey's HSD tests were performed to identify which groups differed significantly. To evaluate whether tourism‐related variables influenced movement behavior, we used Kruskal–Wallis *H*‐tests to compare the frequencies of movement categories (Approach [A], Stationary [S], Walk‐by [W], Partial Retreat [PR], and Full Retreat [FR]) across levels of tourism pressure and tourism‐related variables. Post hoc Mann–Whitney *U* tests were conducted to identify significant pairwise differences. A nonparametric approach was selected because the outcome variable represents ordered categorical data rather than continuous measures, and model assumptions required for parametric analyses (normality and homoscedasticity) were not met.

#### 2.5.2 P2: Tourism‐Related Thresholds

To test the prediction that tourism‐related variables exhibit threshold effects on elephant behavior, we performed segmented (piecewise) linear regressions for each behavioral category. Predictor variables were binned into discrete intervals based on observed data distributions and management‐relevant increments (e.g., 0–50 m, 51–100 m for distance; 0–4, 5–8 for vehicles). Bin intervals were selected to balance sample sizes across categories while representing biologically meaningful or management‐relevant changes in disturbance intensity, consistent with previous studies of wildlife responses to human presence (Ranaweerage et al. 2015; Szott, Pretorius, Ganswindt, and Koyama 2019). Within each bin, mean behavioral rates were calculated to identify potential non‐linear or abrupt shifts. Breakpoints (thresholds) were estimated from segmented regression models as inflection points where behavioral responses changed direction (increased, decreased, or plateaued) beyond the 95% confidence interval of the preceding slope. When assumptions of normality were violated, nonparametric Kruskal–Wallis *H*‐tests followed by pairwise Mann–Whitney *U* tests with Bonferroni correction were used to confirm statistically significant differences among bins. To facilitate management interpretation, we further classified threshold levels as “ideal” (minimal to moderate behavioral change) and “acceptable” (exceeding moderate behavioral change). This combined piecewise and nonparametric approach allowed us to detect and validate threshold responses while ensuring that cutoff values reflected both statistical evidence and ecologically interpretable breakpoints.

#### 2.5.3 P3: Temporal Dynamics

To test whether elephant detection rates varied temporally with park opening and closing transitions, independent *t*‐tests were used to evaluate differences in the mean number of elephants detected per day, number of detection events per day, and mean group size per detection event. This approach was selected because the comparisons involved normally distributed continuous variables summarized by period, and model diagnostics indicated no significant deviations from homogeneity of variance. As these analyses aimed to test overall differences between two discrete conditions (open vs. closed), *t*‐tests provided a straightforward and interpretable method for assessing temporal contrasts. Chi‐squared and Fisher's Exact tests were additionally used to evaluate whether herd‐type composition differed between open and closed periods and across years. The Fisher's Exact test was applied specifically when expected frequencies for certain herd types were below the assumptions of the Chi‐squared test, ensuring valid inference for smaller sample categories.

To account for any gradual changes in elephant behavior over the duration of the study, we included date as a continuous temporal covariate in all models. This variable captured long‐term temporal trends that might arise from factors such as habituation to tourist presence, seasonal variation, or shifts in habitat conditions. The date variable was standardized (mean‐centered and scaled) prior to analyses to facilitate model convergence and comparability of effect sizes across behavioral categories.

### 2.6 Permits and Ethics

All permits necessary for this research have been approved and obtained from the National Research Council of Thailand (NRCT; Permit Number: 104/65), Institutional Animal Care and Use Committee (IACUC: Project ID: KMUTT‐IACUC‐2022/005), and the Department of National Parks, Wildlife and Plant Conservation (DNP; NO: 0401/11523) in Thailand. The research has also been approved by the Chief of Kuiburi National Park, The Kuiburi Wildlife Tourism Group, and the Head of the surrounding Ruam Thai Village.

## 3 Results

Across the 2022–2024 study period, we conducted 113 observation days with 333 elephant detection events, yielding 383 14‐min focal samples and 188 5‐min scan samples during open tourist seasons, and 156 focal samples and 93 scan samples during closed tourist seasons, with an average daily focal sample of 5.05 sessions per day (Table 1).

### 3.1 Behavioral Expression

#### 3.1.1 Variable Influence

**FIGURE 2:**
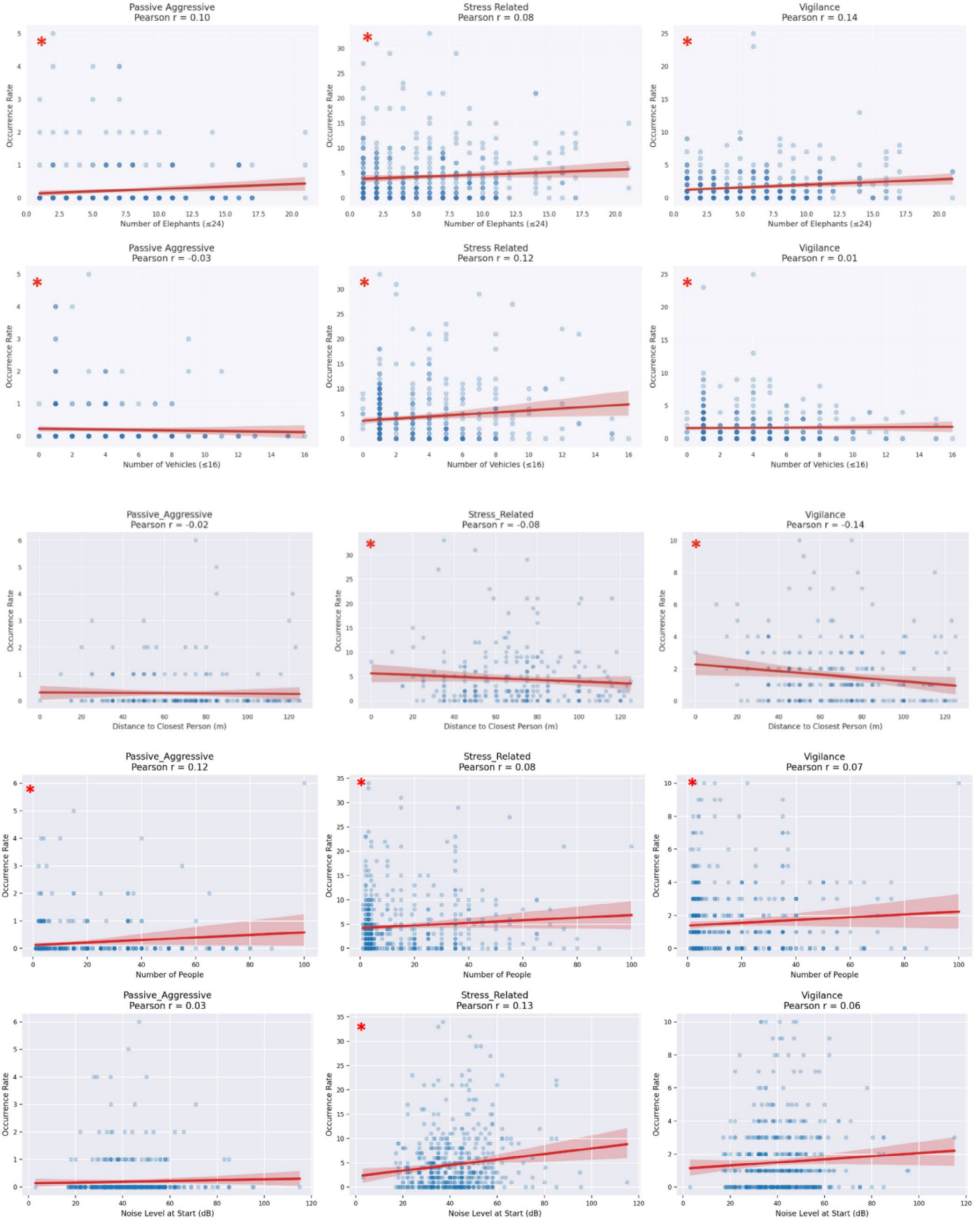
Scatter plots with regression lines showing the relationships between five predictor variables (From top to bottom): Number of elephants, number of vehicles, distance to closest person (m), number of people, and noise level (dB) against three behavioral response occurrences; passive aggressive (left), stress‐related (middle), and vigilance (right). Red lines indicate the linear regression fit, and correlation coefficients (r) are displayed in each panel's title with 95% CI indicated. Statistical significance is indicated by a red asterisk.

The predictor variables with statistically significant influence on overall elephant behavior (*p* < 0.001) included number of vehicles, number of people, noise level, distance to the closest person, date, number of elephants, windy weather, and sex of the elephant (Table 3; Figure 2). The GLMM further revealed distinct patterns in the influence of predictor variables across different behavioral categories (Table 3). For those that achieved statistical significance: STP was positively associated with an increase in passive aggression (*β̂* = 0.4181; *p* < 0.001), stress‐related (*β̂* = 0.0956; *p* < 0.001), and vigilance behavior (*β̂* = 0.1796; *p* < 0.001), and was negatively correlated with affiliative behavior, with the reduction more observed in bulls than cows (*β̂* = −0.372; *p* < 0.001). As distance between elephant and the closest person increased, a slight reduction in stress‐related (*β̂* = −0.0004; *p* = 0.008) and passive aggressive occurrences was observed (*β̂* = −0.005; *p* < 0.001). Conversely, increased distances from elephants to humans increased affiliative behavior (*β̂* = 0.002; *p* < 0.001). Lastly, noise levels increased stress‐related behavior (*β̂* = 0.003; *p* < 0.001).

**TABLE 3:** Summary of Poisson GLMM for all‐occurrence focal sampling behavioral responses with coefficient estimates and *p*‐values for the identified predictor variables with significance across behavioral categories.

Predictor variable	Passive aggression occurrences		Stress‐related occurrences		Vigilance occurrences		Affiliative occurrences	
	*β̂*	*p*	*β̂*	*p*	*β̂*	*p*	*β̂*	*p*
Sighting Tourism Pressure (STP)	4.18 × 10^−1^	**< 0.0001**	9.56 × 10^−2^	**< 0.0001**	1.80 × 10^−1^	**< 0.0001**	−3.72 × 10^−1^	**< 0.0001**
Distance to closest person (m)	−4.90 × 10^−3^	**0.0002**	−4.01 × 10^−4^	**0.0077**	−5.00 × 10^−4^	0.9202	1.70 × 10^−3^	**< 0.0001**
Noise level at start (dB)	−2.40 × 10^−3^	0.6940	3.01 × 10^−3^	**< 0.0001**	−1.60 × 10^−3^	0.3649	−8.60 × 10^−3^	0.0747
Number of elephants	6.01 × 10^−2^	**0.0037**	3.64 × 10^−2^	**< 0.0001**	4.00 × 10^−2^	**< 0.0001**	1.05 × 10^−1^	**< 0.0001**
Sex (Cow)	−2.31 × 10^−2^	0.9146	−8.69 × 10^−2^	**0.0432**	−7.45 × 10^−2^	0.3233	−8.97 × 10^−2^	0.4703
Wind (present/absent)	9.78 × 10^−1^	**0.0024**	7.47 × 10^−2^	0.1026	−1.42 × 10^−1^	0.0921	−3.29 × 10^−1^	**0.0244**
Date (across entire study period)	−2.15 × 10^−8^	**< 0.0001**	−8.22 × 10^−9^	**< 0.0001**	−8.19 × 10^−9^	**< 0.0001**	9.74 × 10^−9^	**0.0006**
Closed‐Open transition (30 days)	7.45 × 10^−2^	**0.0319**	−4.60 × 10^−3^	0.3162	−4.80 × 10^−3^	0.5866	−3.79 × 10^−2^	**0.0222**

*Note:* Predictors include tourism‐related variables and contextual covariates. STP = number of vehicles and number of people (Table 2). Estimates reflect the log change in expected behavior count per unit change in the predictor variable. Positive coefficients indicate an increase in the expected count of the behavior and negative values reflect a decrease. Coefficients (*β*) are presented on a log scale; exponentiated values (exp *β* − 1) approximate percent change in behavioral rate per unit increase in the predictor. While several coefficients are small on a per‐unit basis (distance, noise, group size), they represent cumulative behavioral shifts across ecologically relevant gradients. Bold values indicate statistically significant effects (*p* < 0.05).

The magnitude of several coefficients was small on a per‐unit scale but can reflect meaningful cumulative effects across realistic disturbance gradients when applied at compounding levels. For example, distance to the closest person (*β* = −0.005) indicates a 0.5% decrease in passive‐aggressive behavior per additional meter between elephants and humans, translating to a 25%–40% decrease across typical 50–100 m distance increases. Similarly, the coefficients for number of people (*β* = 0.0047) and noise level (*β* = 0.003) correspond to slight per‐unit increases (~0.5%–0.3%); however, they can accumulate more impactfully at high disturbance levels (> 20 tourists; > 40 dB). These effects align with the threshold patterns observed in subsequent analyses.

For non‐tourism related variables, the number of elephants was a significant predictor across behaviors (Table 3), with the strongest effect observed on affiliative (*β* = 0.1050; *p* < 0.001), passive aggressive (*β* = 0.0601; *p* = 0.004), vigilance (*β* = 0.0400; *p* < 0.001) and stress‐related behaviors (*β* = 0.0364; *p* < 0.001). Windy conditions were associated with reduced affiliative behavior (*β* = −0.329; *p* = 0.024), and increased passive aggressive occurrences (*β* = 0.978; *p* = 0.002). For temporal factors, park transition (the shift in park status from closed to open) exerted its strongest influence on passive aggression (*β* = 0.0745; *p* = 0.032), with elevated levels observed immediately after park opening while affiliative behavior declined after park opening (*β* = −0.0379; *p* = 0.022).

Generalized Linear Mixed Model (GLMM) comparisons revealed that tourism‐related variables were consistently the strongest predictors of behavioral responses to disturbance (Table 4). The number of vehicles was the strongest tourism‐related predictor across models. It significantly increased passive‐aggressive (*β* = 0.039; *p* = 0.0001; *R* ^2^ = 0.047; Table 4), stress‐related (*β* = 0.238; *R* ^2^ = 0.022; *p* = 0.0007), and vigilance behaviors (*β* = 0.077, *p* = 0.0025, *R* ^2^ = 0.017). The number of people also positively influenced passive‐aggressive (*β* = 0.0047; *p* = 0.002) and stress‐related behavior (*β* = 0.035; *p* = 0.0097), although its explanatory power was lower (*R* ^2^ = 0.018 and 0.013, respectively). Distance to the closest person was negatively associated with passive‐aggressive responses (*β* = −0.0006; *p* = 0.0044), indicating greater aggression when people were closer. Noise level at the start of observations was a weaker predictor, though it significantly affected stress‐related behaviors (*β* = 0.021; *p* = 0.018). After controlling for spatial effects such as distance of elephant to closest person, analyses continued to reveal significant behavioral responses to tourism‐related variables. For vigilance, the number of vehicles remained a significant positive predictor despite distance (*β* = 0.135, *p* = 0.002) as well as for passive‐aggressive behaviors (*β* = 0.318, *p* = 0.007).

**TABLE 4:** Summary of GLMM results evaluating the influence of tourism‐related variables on three elephant behavioral response categories. The non‐anthropogenic variables included in the AIC analysis are those that were identified as statistically significant in the initial GLMM (Table 3).

Behavior category	Tourism‐related variable	AIC	ΔAIC	*R* ^2^	*p*	*β* coefficient
Passive aggressive	Number of Vehicles	1005.94	0.00	0.047	**0.0001**	0.0390
	Number of People	1021.54	15.60	0.018	**0.0020**	0.0047
	Distance To Closest Person (m)	1023.03	17.09	0.015	**0.0044**	−0.0006
	Noise Level At Start (dB)	1031.16	25.22	0.000	0.9543	−0.0001
Stress‐related	Number of Vehicles	3309.90	0.00	0.022	**0.0007**	0.2383
	Number of People	3314.84	4.95	0.013	**0.0097**	0.0354
	Noise Level At Start (dB)	3315.95	6.06	0.011	**0.0182**	0.0207
	Distance To Closest Person (m)	3320.70	10.80	0.002	0.3543	0.0019
Vigilance	Number of Vehicles	2254.71	0.00	0.017	**0.0025**	0.0770
	Number of People	2260.82	6.12	0.006	0.0804	0.0087
	Noise Level At Start (dB)	2263.82	9.12	0.000	0.7915	−0.0008
	Distance To Closest Person (m)	2263.84	9.13	0.000	0.8121	−0.0002

Response	Non‐anthropogenic variable	AIC	ΔAIC	*R* ^2^	*p*	*β* coefficient
Passive aggressive	Date (ordinal)	1022.42	0.00	0.017	**0.0032**	−0.0003
	Number of elephants	1028.95	6.53	0.004	0.1380	0.0089
Stress‐related	Number of elephants	3311.31	0.00	0.019	**0.0014**	0.1703
	Date (ordinal)	3313.78	2.47	0.015	**0.0054**	−0.0023
Vigilance	Date (ordinal)	2254.54	0.00	0.018	**0.0023**	0.0592
	Number of elephants	2256.89	2.35	0.016	**0.0042**	−0.0007

*Note:* Predictors include number of vehicles, number of people, distance to the closest person (m), and noise level at the start of observation (dB). For each model, Akaike's Information Criterion (AIC), change in AIC (ΔAIC), coefficient of determination (*R* ^2^), statistical significance (*p*‐value), and *β* coefficient are reported. Statistically significant predictors (*p* < 0.05) are shown in bold. Models are ranked within each behavior type by AIC.

Among non‐anthropogenic variables, date and number of elephants both significantly improved model fit across behaviors (Table 4). Models including date had lower AIC values (ΔAIC = 0–2.47) than those with number of elephants (ΔAIC = 2.35–6.53), indicating stronger overall support for temporal effects. Although coefficients were small (|*β*| ≤ 0.0023), date was significant for all behaviors (*p* < 0.01) and explained comparable variance in several tourism‐related predictors (*R* ^2^ = 0.015–0.019), suggesting gradual declines in stress, vigilance, and aggression over time, and slight increases in affiliative behavior. The number of elephants also improved model performance for stress‐related and vigilance behaviors (ΔAIC ≤ 2.35; *p* < 0.01), with larger groups showing higher behavioral activity. Together, these results indicate that temporal dynamics and group context provided better model fits than other environmental factors, highlighting their influence alongside tourism‐related predictors.

#### 3.1.2 Activity Budget

From the activity budget analyses, a specific vigilance behavior duration state of “alert” (in seconds; Table S1) increased with increasing STP (*H* = 20.16, *p* < 0.001). Flee/Retreat behavior significantly increased with increasing STP (*H* = 12.82, *p* = 0.005) where the duration of retreat behavior increased with tourism pressure, with the highest mean durations observed under Extreme pressure. Pairwise Mann–Whitney *U* tests with Bonferroni correction revealed that elephants were significantly “alert” for longer durations under an STP of High compared to Medium (*U* = 2123.5; *p* = 0.001), Low (*U* = 874; *p* = 0.004), and None (*U* = 1424; *p* = 0.049). Flee/Retreat behavior significantly increased with increasing STP (*H* = 12.82, *p* = 0.005), with the highest mean durations observed under Extreme pressure.

#### 3.1.3 Demographic Influence

**FIGURE 3:**
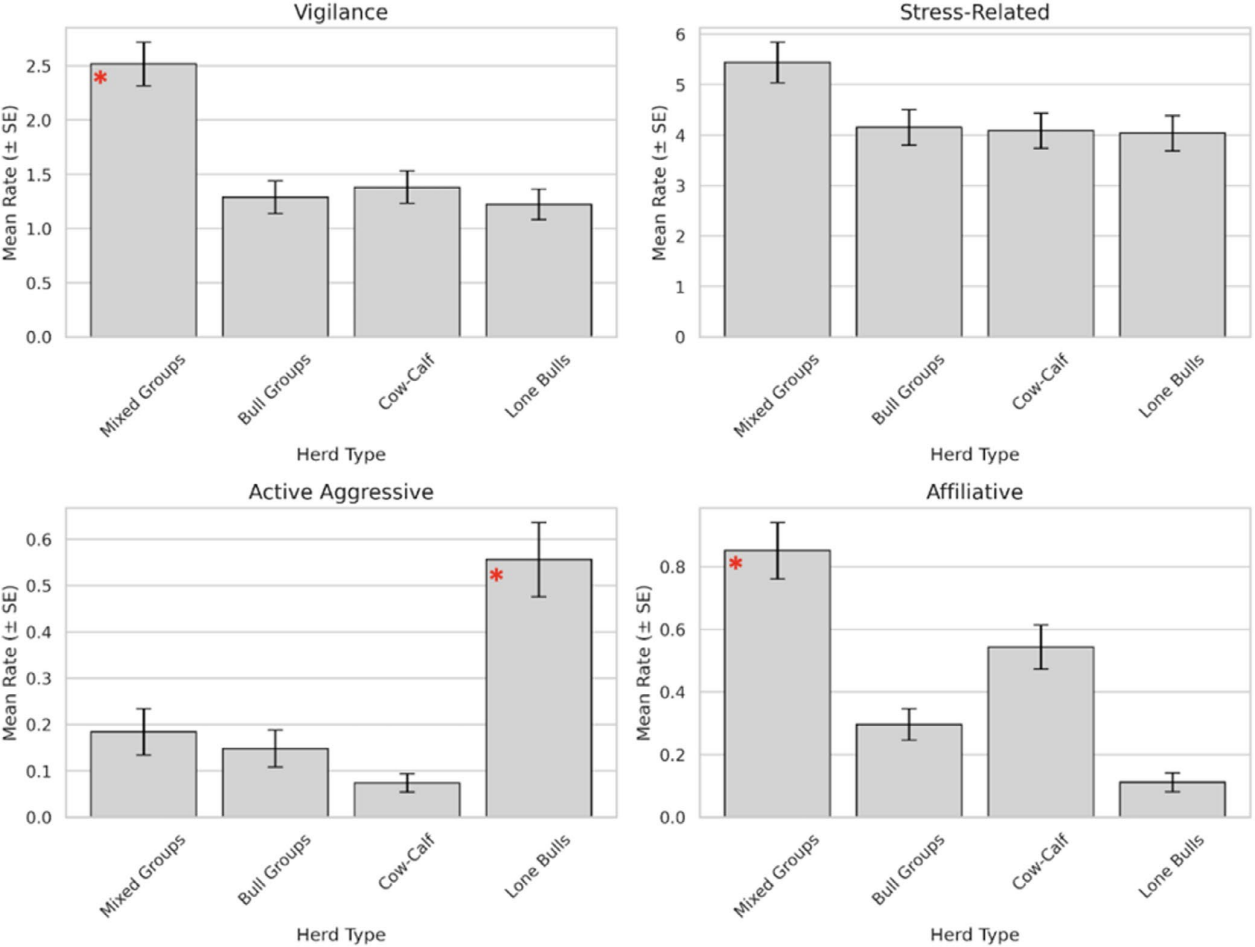
Top left: Mean behavioral rates (± SE) across elephant herd types for vigilance behavior. Statistically significant differences are indicated with an asterisk. Top right: Mean behavioral rates (± SE) across elephant herd types for stress‐related behavior. Bottom left: Mean behavioral rates (± SE) across elephant herd types for active aggressive behavior. Statistically significant differences are indicated with an asterisk. Bottom right: Mean behavioral rates (± SE) across elephant herd types for affiliative behavior.

One‐way ANOVA analyses revealed significant effects of herd type on several behavioral outcomes (Figure 3). Active aggression differed significantly across herd types, with Lone bulls exhibiting higher rates than Mixed, Bull, and Cow‐calf groups (*p* = 0.004). Vigilance behavior also varied significantly by herd type (*p* < 0.001), with Mixed groups showing higher vigilance levels than Bull and Cow‐calf groups. Affiliative behavior was significantly influenced by herd type (*p* = 0.002), peaking in Mixed groups (*x̄* = 0.851), which differed significantly from Lone bulls and Cow‐calf groups (Tukey's HSD, *p* < 0.05).

#### 3.1.4 Movement Patterns

**FIGURE 4:**
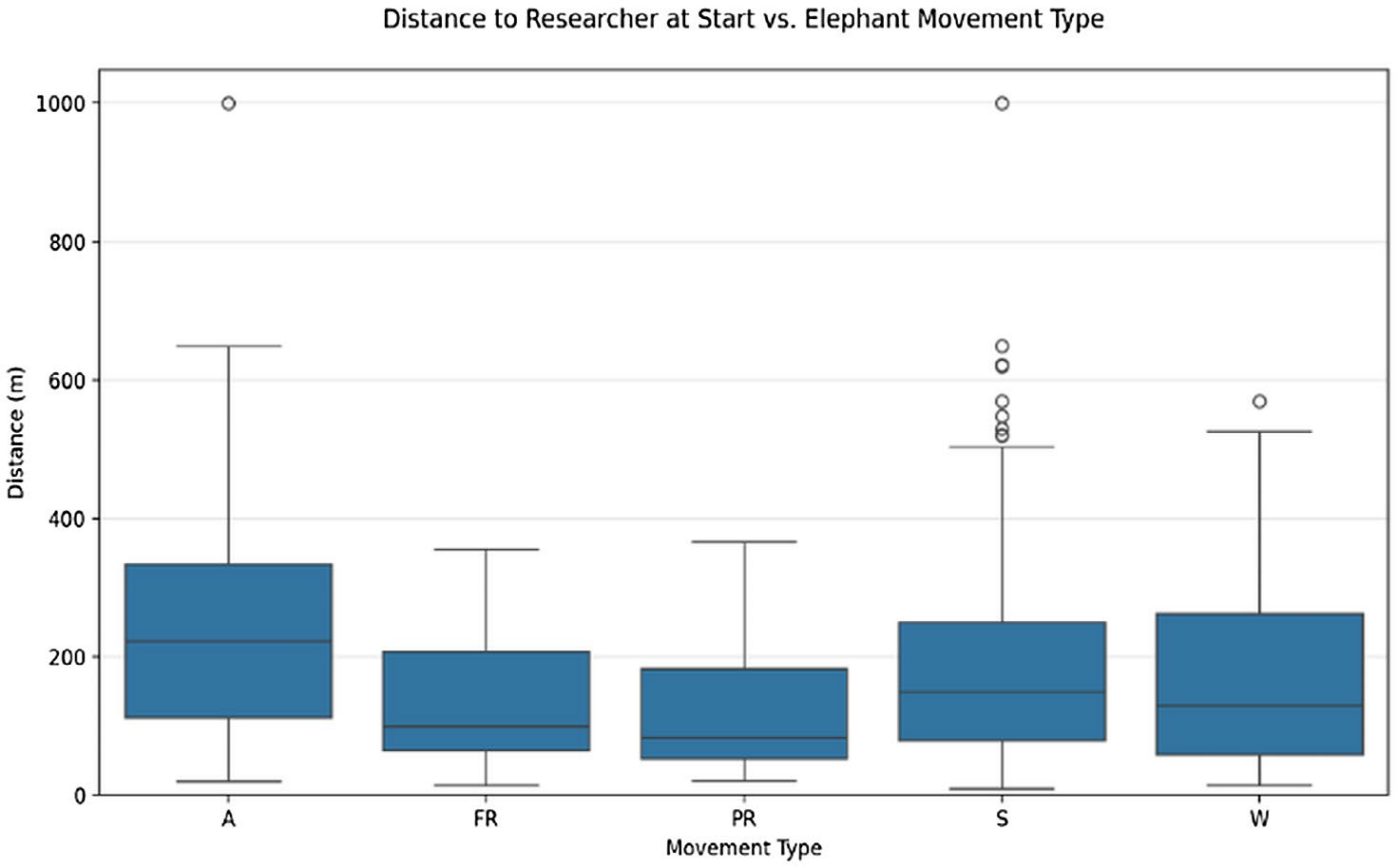
Distance from closest human at the start of observation across different elephant movement types. Boxplots represent the median, interquartile range, and outliers for each movement category: Approach (A), Full Retreat (FR), Partial Retreat (PR), Stationary (S), or Walk‐by (W).

A significant effect of distance of closest human to elephant on movement type patterns (A, S, W, PR, FR) during the five‐minute scan sampled was detected using a Kruskal–Wallis *H*‐test (*H* = 15.23; *p* = 0.004; Figure 4). Post hoc Mann–Whitney *U* tests revealed that elephants exhibiting Partial Retreat (PR) behavior were significantly closer to humans than those that remained Stationary (S) (*p* < 0.001). Further, Stationary, Approach, and Walk‐by behaviors overall were associated with greater median distances from humans as opposed to Partial Retreat and Full Retreat (Figure 4).

Noise levels were also shown to have a significant influence on movement type (*H* = 11.84; *p* = 0.019). Post hoc Mann–Whitney *U* tests showed that Full Retreat (FR) was associated with higher noise levels compared to both Stationary (S) (*p* = 0.013) and Partial Retreat (PR) (*p* = 0.003). When Full and Partial Retreats were pooled into a single category, elephants in the Retreat group were found to be exposed to significantly higher noise levels than those in the Stationary group (*p* = 0.003).

### 3.2 Tourism‐Related Thresholds

#### 3.2.1 Number of Vehicles

**FIGURE 5:**
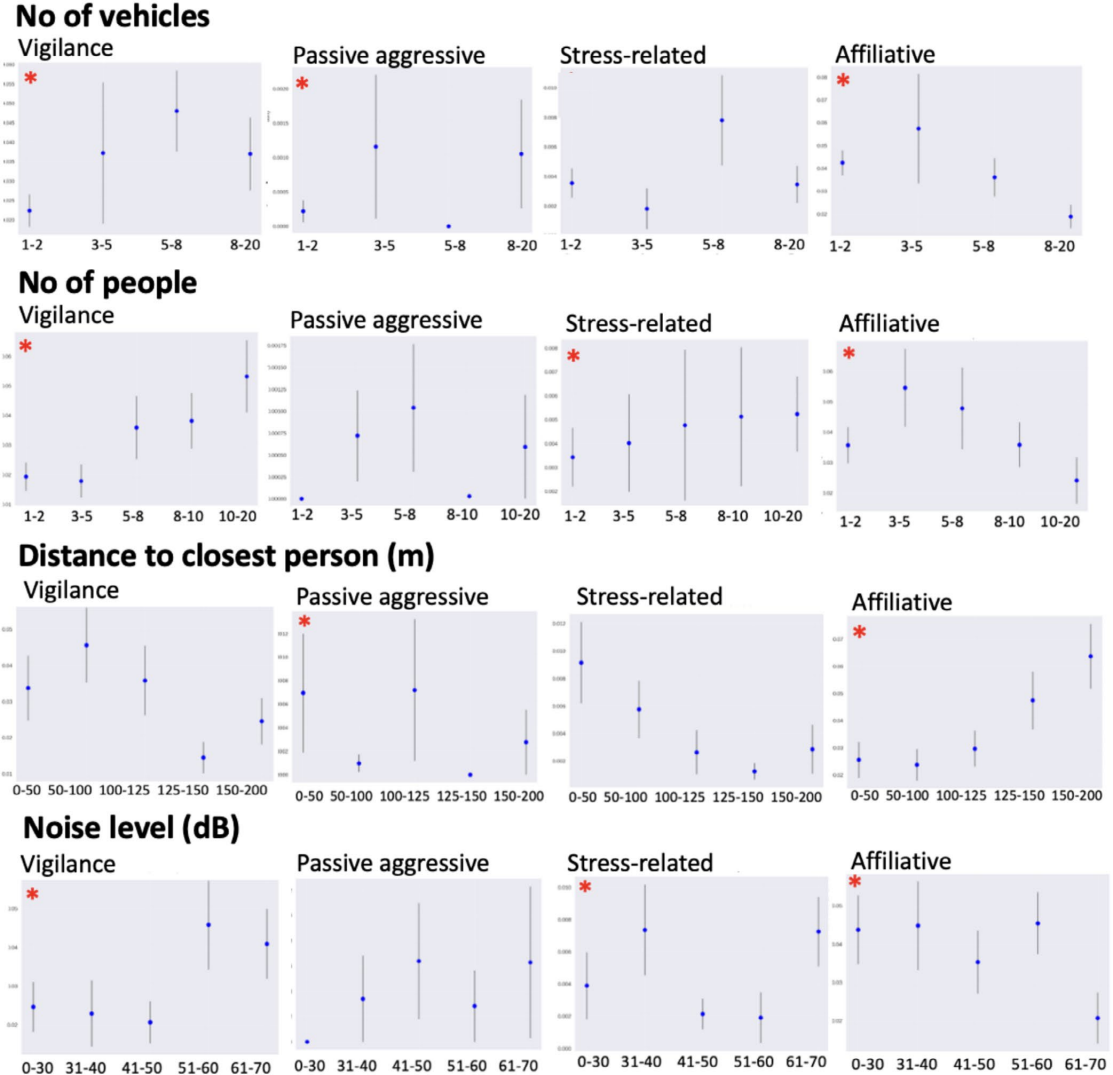
Threshold analysis of elephant behavioral responses to tourism‐related predictors, grouped by predictor variable. Each panel shows mean behavioral rates (±SE) for four behavioral categories (vigilance, passive‐aggressive, stress‐related, and affiliative) in relation to binned levels of (top to bottom) number of vehicles, number of people, distance to the closest person, and ambient noise level (dB). Red asterisks (*) indicate statistically significant differences across bins (Kruskal–Wallis *H*‐tests, *p* < 0.05). Although the total sample size was consistent (*n* = 523), the number of observations per quantile bin varied across predictors. Noise level and distance to the closest person had similar bin sizes (~130), while number of people and vehicles showed greater variability (64–301), contributing to differences in error estimation magnitudes due to both sample size and within‐bin variability.

Passive aggressive (*H* = 19.78; df = 4; *p* = 0.00055) and vigilance (*H* = 14.76; df = 4; *p* = 0.011) behavior responses indicated a statistically significant threshold shift beyond four and eight vehicles and remained consistently elevated at high vehicle densities (Figure 5). Whereas affiliative behavior declined significantly beyond four vehicles (*H* = 11; df = 4; *p* = 0.0265).

#### 3.2.2 Number of People

Vigilance behaviors showed a statistically significant stepwise increase with larger tourist groups, particularly beyond 10 and 20+ individuals (*H* = 14.5138, df = 5; *p* = 0.0126). Stress‐related behaviors also showed a significant increase (*H* = 11.998, df = 5; *p* = 0.0348) beyond 10 individuals. And moderate to large groups (10+ individuals) significantly decreased affiliative behaviors (*H* = 12.2032, df = 5; *p* = 0.0321) with a decline initiating at as little as four people (Figure 5).

#### 3.2.3 Distance to Closest Person

Significant increases in passive aggressive behaviors were observed within the 0–50 m range (*H* = 14.5808, df = 5; *p* = 0.0123) and were reduced beyond 100 m (Figure 5), while stress‐related behaviors increased sharply at distances under 50 m. Conversely, the highest rates of affiliative behavior occurred at distances greater than 200 m from tourists with a threshold identified at 151 m (*H* = 12.7718, df = 5; *p* = 0.0256). Vigilance behavior rates were highest at close proximities (< 50 m) and decreased with increasing distances, stabilizing beyond 100 m.

#### 3.2.4 Noise Level

Ambient noise levels exceeding 40–50 dB increased vigilance, passive aggressive, and stress‐related behaviors (*H* = 11.9249, df = 5; *p* = 0.0495) with affiliative behavior declining sharply above 50 dB (*H* = 14.76, df = 5; *p* = 0.0114).

Overall, behaviors significantly increased once environmental pressures exceeded specific thresholds (Table 5) with “ideal” and “acceptable” thresholds identified (≥ 32 dB/≥ 40 dB), (≥ 10/≥ 21 people), (≥ 4/≥ 8 vehicles), and (≤ 100/≤ 125 m from people) identified. The points at which we see a substantial and abrupt behavior change occurred at ≥ 50 dB, ≥ 21 people, ≥ 10 vehicles, and within 50 m. Affiliative behavior demonstrated more sensitive thresholds: ≥ 40 dB, ≥ 4 vehicles, ≥ 6 people, and ≤ 200 m distance. At the highest thresholds, there can be seen a reduction in behaviors likely due to retreat and other avoidance behaviors taking over as the secondary response to disturbance.

**TABLE 5:** Threshold points for changes in elephant behavior in response to tourism‐related factors.

Behavior	Environmental factor	Threshold point (Ideal)	Threshold point (Acceptable)	% behavior change acceptable to ideal
Passive aggressive	Noise Level (dB)	40	50	−5.27
Passive aggressive	Number of Vehicles	2	4	−1.43
Passive aggressive	Number of People	10	10	−14.34
Passive aggressive	Distance to People (m)	125	100	−20.54
Stress‐related	Noise Level (dB)	40	50	−13.47
Stress‐related	Number of Vehicles	4	8	−2.06
Stress‐related	Number of People	10	20	−4.41
Stress‐related	Distance to People (m)	125	100	−3.79
Vigilance	Noise Level (dB)	32	50	−27.84
Vigilance	Number of Vehicles	2	4	−7.88
Vigilance	Number of People	6	10	−5.55
Vigilance	Distance to People (m)	125	100	0.51
Affiliative	Noise Level (dB)	32	50	39.07
Affiliative	Number of Vehicles	2	4	7.54
Affiliative	Number of People	6	20	13.99
Affiliative	Distance to People (m)	200	151	1.06

*Note:* Values represent the minimum point at which a notable directional change (increase or decrease) in the rate of passive aggressive, stress‐related, vigilance, and affiliative behaviors was observed and the thresholds for “ideal” and “acceptable” behavior change as well as the percent change in behavior from acceptable to ideal and its direction.

The comparison of ideal to acceptable thresholds revealed distinct behavioral shifts where increased viewing distance elicited a 20% decrease in passive aggressive behavior from “acceptable” to “ideal” conditions (Table 5). Elevated noise levels had a dual impact by significantly decreasing by 27% and stress‐related behaviors by 13%, while concurrently increasing affiliative behaviors by 39%. The reduction in vehicle numbers between acceptable to ideal showed a 7% reduction in vigilance and a 7% increase in affiliative behavior, and the number of people from “ideal” to “acceptable” conditions decreased passive aggressive behavior by 14% with a concurrent 14% increase in affiliative behavior.

Identifying Daily Tourism Pressure thresholds was less definitive. Vigilance behaviors significantly increased from None to Extreme DTP (*U* = 12,812; *p* = 0.0051). Notably, affiliative behavior decreased by ~16% beyond a DTP of Medium (*p* = 0.002) and passive aggressive behavior increased by roughly 76% beyond a tourism pressure of Low, although not statistically significant (*p* = 0.484) due to high variability in the data.

### 3.3 Temporal Dynamics

**FIGURE 6:**
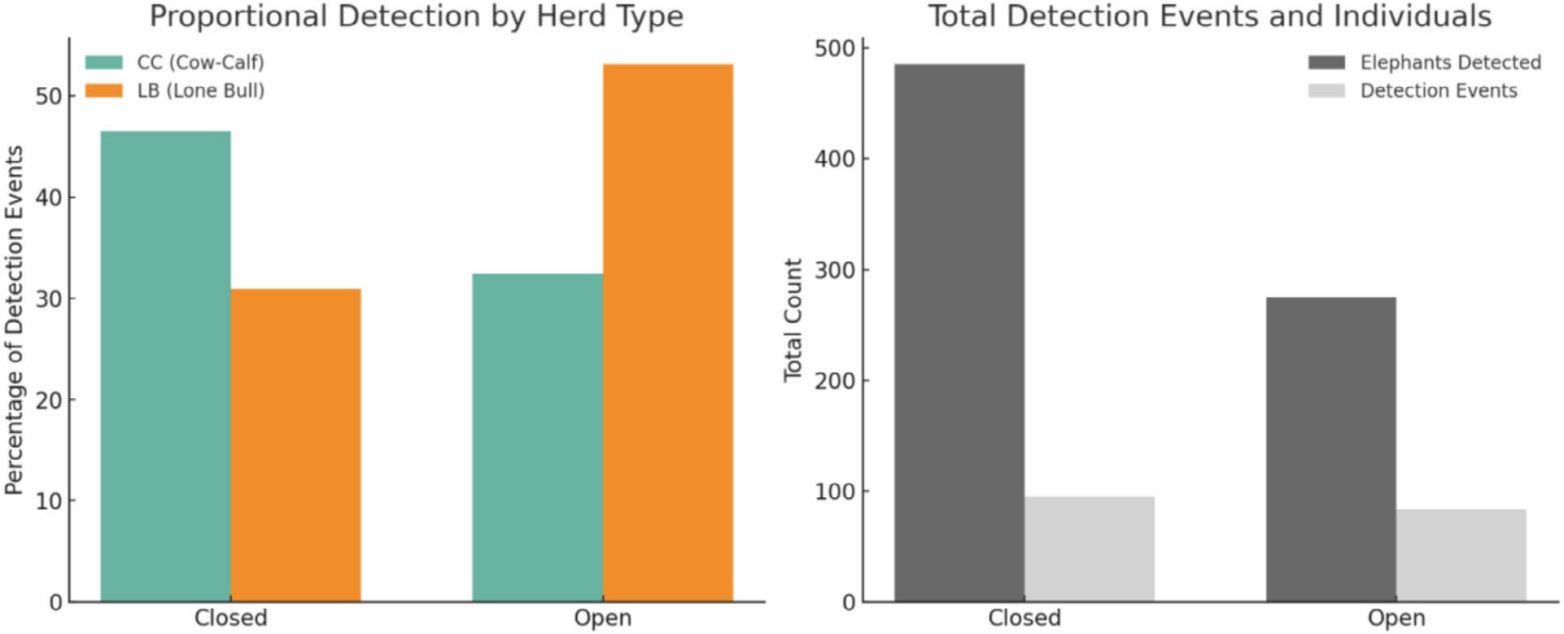
Left: Proportional detection events by herd type CC = Cow‐calf group and LB = Lone Bulls during open and closed periods. Right: Absolute totals of both elephants detected and detection events during open and closed periods; pooled across 2022 and 2023.

A comparison of elephant detections between open and closed seasons (2022 and 2023) revealed statistically significant patterns in the mean elephants detected per day (daily total of elephants), which was significantly higher when the park was closed (*x̄* = 16.80) compared to when it was open (*x̄* = 7.70), confirmed by a *t*‐test (*t* = 2.72; *p* = 0.001; Figure 6). The mean number of detection events per day dropped significantly from an average of 3.34 during closed periods to 2.17 during open periods (*t* = 2.80; *p* = 0.008), and the mean number of elephants per detection event was significantly higher (*t* = 2.26; *p* = 0.025) during closed periods (*x̄* = 5.03) compared to open periods (*x̄* = 3.54; Figure 6).

A Chi‐squared test revealed a significant difference between open and closed periods for herd type (*χ* ^2^ = 4.14; *p* = 0.042), confirmed by a Fisher's Exact Test (*p* = 0.031; Figure 6). Cow‐calf groups were detected more frequently during closed periods, while Lone bulls were more commonly observed when the park was open (*χ* ^2^ = 9.74; *p* = 0.021; Figure 6). Following the transition from closed to open in 2022 (October–November), CC detections declined by 8%, while LB detections increased by 46% and in the 2023 transition period (June–July), CC detections dropped by 61% and LB detections increased by 24%. Proportional detection events by herd type show that CC groups represented a greater proportion of detections during closed periods (46%) compared to open periods (32%), while LB's exhibited the opposite pattern, increasing from 31% (closed) to 53% (open). Absolute totals indicate a higher number of both elephants detected and detection events during closed periods (Figure 6).

Passive aggression (*β* = −2.15 × 10^−8^; *p* < 0.001; Table 4), stress‐related (*β* = −8.22 × 10^−9^; *p* < 0.001), and vigilance behaviors (*β* = −8.19 × 10^−9^; *p* < 0.001) all declined slightly over time, while affiliative behaviors increased marginally (*β* = 9.74 × 10^−9^; *p* = 0.0006). Active aggression also increased modestly (*β* = 3.20 × 10^−9^; *p* = 0.034). Although these effect sizes were small, the consistent directions suggest a gradual reduction in stress and vigilance and a mild increase in social and aggressive interactions over the study period.

## 4 Discussion

The overall hypothesis and related predictions (P1, P2, P3) tested during the course of the research were well supported by our findings and offer clear evidence for guidelines for wild elephant tourism.

First, our findings support our overall hypothesis including P1, where elevated tourism pressure increases stress‐related, vigilance, and passive aggressive behaviors, while reducing affiliative behaviors and altering movement patterns. Overall, tourism‐related variables and pressures significantly increased rates of passive aggression, stress‐related, and vigilance behaviors, and decreased affiliative behaviors, particularly under High and Extreme tourism pressure conditions (Table 2) with affiliative behaviors declining even under Low pressure, suggesting social interactions are highly sensitive to human disturbance (Szott 2020). Daily Tourism Pressure was less influential than STP, but similarly showed a negative correlation with affiliative behavior and positive influence on stress‐related and passive aggression. Overall, elephants exhibited behavior‐specific sensitivities to both cumulative tourism pressure (DTP) and immediate human disturbance (STP), highlighting the necessity to manage both forms of disturbance at sighting specific and park entry levels. Further, affiliative thresholds emerged as a specific concern. While vigilance and passive aggressive behavior increased with tourist exposure—simultaneously, affiliative interactions were reduced, possibly as a trade‐off between social bonding and risk management. Affiliative behaviors were more maintained in females under increasing tourism pressure, suggesting that behaviors significant for males such as sparring, play, and mating may be at greater risk of interruption. Due to their sensitivity, affiliative behaviors may serve as an early indicator of disturbance and should be weighed more heavily in management recommendations (Table 6).

**TABLE 6:** Thresholds and management recommendations for key tourism‐related predictors influencing elephant behavior.

Predictor	Behavior category	Thresholds (Ideal/Acceptable)	Management recommendation
Distance to Closest Person (m)	Passive aggressive	> 125 m/> 100 m	Maintain distances > 125 m, minimum acceptable > 100 m.
	Stress‐related	< 125 m/100 m	Maintain distances > 125 m, minimum acceptable > 100 m.
	Vigilance	< 125 m/100 m	Maintain distances > 125 m, minimum acceptable > 100 m.
	Affiliative	< 200 m/125 m	Preference for sightings at ~200 m for CC & MG.
Number of People	Passive Aggressive	> 10 ppL/< 20 ppL	Limit sightings to ideally ≤ 10, acceptably ≤ 21 people.
	Stress‐related	> 10 ppl/20 ppl	Limit sightings to ideally ≤ 10, acceptably ≤ 21 people.
	Vigilance	> 10 ppl/20 ppl	Limit sightings to ideally ≤ 10, acceptably ≤ 21 people.
	Affiliative	> 6 ppL/10 ppl	Keep to ≤ 10 people, ideally 6 for CC & MG.
Number of Vehicles	Passive aggressive	> 4 veh./8 veh.	Restrict to ideally ≤ 4, acceptable ≤ 8 vehicles per sighting.
	Stress‐related	> 4 veh./8 veh.	Restrict to ideally ≤ 4, acceptable ≤ 8 vehicles per sighting.
	Vigilance	> 4 veh./8 veh.	Restrict to ideally ≤ 4, acceptable ≤ 8 vehicles per sighting.
	Affiliative	> 2 veh./4–8 veh.	Limit vehicles ideally ≤ 2; maximum 4–8 for CC & MG.
Noise Level (dB)	Passive aggressive	< 32 dB/< 40 dB	Maintain noise levels ideally < 32 dB, acceptably < 40 dB.
	Stress‐related	> 32 dB/40 dB	Maintain noise levels ideally ≤ 32 dB, acceptably ≤ 40 dB.
	Vigilance	> 32 dB/40 dB	Maintain noise levels ideally ≤ 32 dB, acceptably ≤ 40 dB.
	Affiliative	> 40 dB/50 dB	Maintain noise levels ideally ≤ 42 dB.

*Note:* Ideal and acceptable limits are shown for each predictor and behavioral category, providing evidence‐based management recommendations for minimizing disturbance during wildlife tourism.

For herd type behavior differences, active aggression was highest in lone bulls, while vigilance and affiliative behaviors peaked in mixed groups, suggesting that social composition shapes behavior and arousal, with Mixed groups exposed to more complex social dynamics (i.e., reproductive activity). Therefore, incorporating herd type into tourism management for groups with increased sensitivity may reduce behavioral disruptions through context‐specific welfare strategies. Limiting viewing groups (people and vehicles) and maintaining greater distances and reduced noise levels with mixed and cow‐calf groups are thus recommended for management.

Ordinal date and elephant group size were the most important non‐tourism‐related predictors. The number of elephants was a consistent predictor across all behavioral categories, with larger elephant groups associated with increased active aggression, stress‐related, and affiliative behavior. Interestingly, active aggressive behaviors declined when the number of tourists exceeded the number of elephants, potentially reflecting a perception by elephants of a numerical disadvantage triggering an avoidance strategy; although more study would be needed to confirm this.

Over time, stress‐related and vigilance behaviors declined, but were accompanied by increases in active aggression, suggesting habituation and highlighting a complex adaptive dynamic that requires careful monitoring. Immediately after park re‐opening following closure periods, passive aggression increased and affiliative behavior declined, suggesting that transitions in disturbance regimes trigger acute responses and may lead to short‐term behavioral disruptions characterized by increased arousal and withdrawal. The pattern is consistent with a habituation reset effect, in which elephants may respond more strongly to human presence following periods of respite from tourism activity (Shelton and Higham 2007; Gaynor et al. 2020). These findings highlight the need for regulated tourism regimes that include gradual phasing of park closure and opening periods to reduce heightened arousal. Our findings also showed evidence of behavioral plasticity across tourism periods, suggesting habituation; however, habituation may mask underlying physiological stress that behavioral data cannot capture (Bateman and Fleming 2017; Chock et al. 2025). Due to the possibility of habituation to exert negative and unintended consequences (Higham and Shelton 2011; Geffroy et al. 2015; Bateman and Fleming 2017), we encourage future research on physiological stress sampling (i.e., dung sampling for stress hormones) to further assess physiological responses.

Our findings further support that certain tourism‐related variables would exert stronger influences on behavior than others, with specific thresholds (P2) triggering behavioral shifts, also supported. In order of statistical influence: number of vehicles, number of people, distance to humans, noise level, number of elephants, and date emerged as the most significant predictors of behavioral responses. Overall, the number of vehicles was the most influential in passive aggression, stress‐related, and vigilance behaviors, with model selection reinforcing the primacy of vehicles in predicting behavior, with strong explanatory power across behavioral categories. This suggests that vehicle presence not only generates visual and acoustic disturbance but can contribute to crowding and risk perception, as found in other studies (Hsu et al. 2025).

The number of people was also substantial in predicting stress‐related behavior and altering movement patterns as well as distances (< 100 m) between elephants and humans initiating increased stress‐related and passive aggressive behaviors and reduced affiliative interactions, also seen in other studies (LaDue et al. 2025). After accounting for spatial variables, tourism‐related variables continued to significantly predict behavioral changes, particularly vigilance and passive aggression. These findings suggest that while proximity plays a role, it does not fully account for all behavioral variation, with other tourism variables contributing independently and underscores the importance of visitor management strategies even within relatively consistent spatial contexts. Noise levels significantly increased stress‐related behaviors, reinforcing that ambient acoustic disturbance can have significant impacts on wildlife behavior (Quadros et al. 2014).

Our findings further indicate that elephant movement responses were significantly influenced by human proximity, with elephants engaging in retreat behaviors at closer distances to humans. Retreat behavior was also associated with higher noise levels—corroborating the role of spatial proximity and acoustic disturbance in elephant response to human presence (Ruesto et al. 2010; Quadros et al. 2014; Muntifering et al. 2019; Arcangeli et al. 2022). This, in turn, in addition to altering their natural movements, may impact tourism by reducing detectability and viewing time.

Statistically significant behavioral thresholds (P2) in response to tourism pressure were identified in both the intensity and type of disturbance. From this, several management recommendations for wild elephant viewing were developed to reduce tourism‐related impacts (Table 6; Figure 5). Our findings indicate that reduced human and vehicle numbers, quiet observational practices, and maintaining moderate viewing distances are critical in reducing behavioral stress responses in elephants and maintaining elephant social cohesion. While distance to the closest person and noise level are important, their impacts are often intertwined with those of vehicle/human presence, suggesting that managing vehicle/human numbers could directly alleviate other impacts. Affiliative behavior emerged as the most sensitive indicator of tourism disturbance with the strictest thresholds. Decline in affiliative behaviors, which are essential for social cohesion and welfare, is consistent with other studies (Szott 2020; Patterson et al. 2024) and the clear suppression of affiliative behaviors observed, even at low tourism levels, underscores the need for deliberate tourism management using the more sensitive thresholds (Larson et al. 2016).

Comparative analyses of “ideal” versus “acceptable” tourism conditions demonstrated that even small changes in viewing distance, noise level, number of people, and number of vehicles yielded meaningful behavioral improvements. For instance, reducing noise by 10 dB decreased stress‐related behavior by 13% and vigilance by 27%, while affiliative behavior increased by 39%. Highlighting significant welfare gains through modest reductions in sighting tourism pressure again demonstrates that the heightened sensitivity of affiliative behavior indicates different herd types require different considerations, with cow‐calf and mixed group viewing better conducted under “ideal” conditions.

Lastly, our findings support the prediction (P3) that behavioral responses and detection rates vary temporally, with park opening and closing transitions. Open and closed tourism seasons demonstrated significant differences in detection frequency, with more elephants detected during park closures, including higher number of detection events overall and more elephants observed per event. Herd type also differed, with cow‐calf groups detected significantly less frequently during open periods; conversely, bulls were detected more frequently. This highlights that detection of herd types is likely influenced by tourism pressure and thus reduces detectability of more vulnerable herd types, such as cow‐calf groups during higher tourism intensity. Socially cohesive groups engaging in spatial or temporal avoidance strategies to avoid human disturbance could be compromising their access to critical resources or disrupting natural processes (Chaiyarat et al. 2024; Patterson et al. 2025). To prevent this, tourism management that minimizes disturbance around key resources, such as waterholes and grazing areas, along with increased support for vulnerable social groups, like breeding herds, should be incorporated (Piñeiro et al. 2012; Larson et al. 2016).

Finally, some unavoidable limitations of the research should be acknowledged. Elephant detections were based on daily road surveys that prioritized behavioral data collection rather than systematic coverage and do not permit inferences about occupancy or population size. The timing and duration of research efforts varied between years due to park‐level logistical changes, which may have incurred slight differences in seasonal weather patterns. Certain behavioral categories, such as active aggression, were observed infrequently, resulting in small sample sizes that were unable to be used consistently. Certain variables could shift over the course of the 14‐min observation period (i.e., noise level, number of elephants), introducing additional variability to the dataset.

The inability to identify individual elephants reduced individual variation control; particularly in repeated‐use areas (English et al. 2014). Finally, because observers were present during all data collection, including during park closure periods, some human presence was unavoidable. Future research should aim to build on these findings by incorporating longitudinal and multi‐seasonal data, particularly during the dry season when elephants may be more concentrated near water sources. The inclusion of individual identification methods (i.e., photo‐ID, AI‐based recognition) would allow for assessment at the individual level and integrating physiological indicators (e.g., fGCM in dung) could validate behavioral responses. Future studies should also explore indirect or cascading impacts of tourism, such as changes in spatial use patterns, altered foraging behavior, or long‐term reproductive success and could incorporate the use of video cameras at viewing sites to enhance the behavioral monitoring of tourist–elephant interactions.

### 4.1 Management Recommendations

**FIGURE 7:**
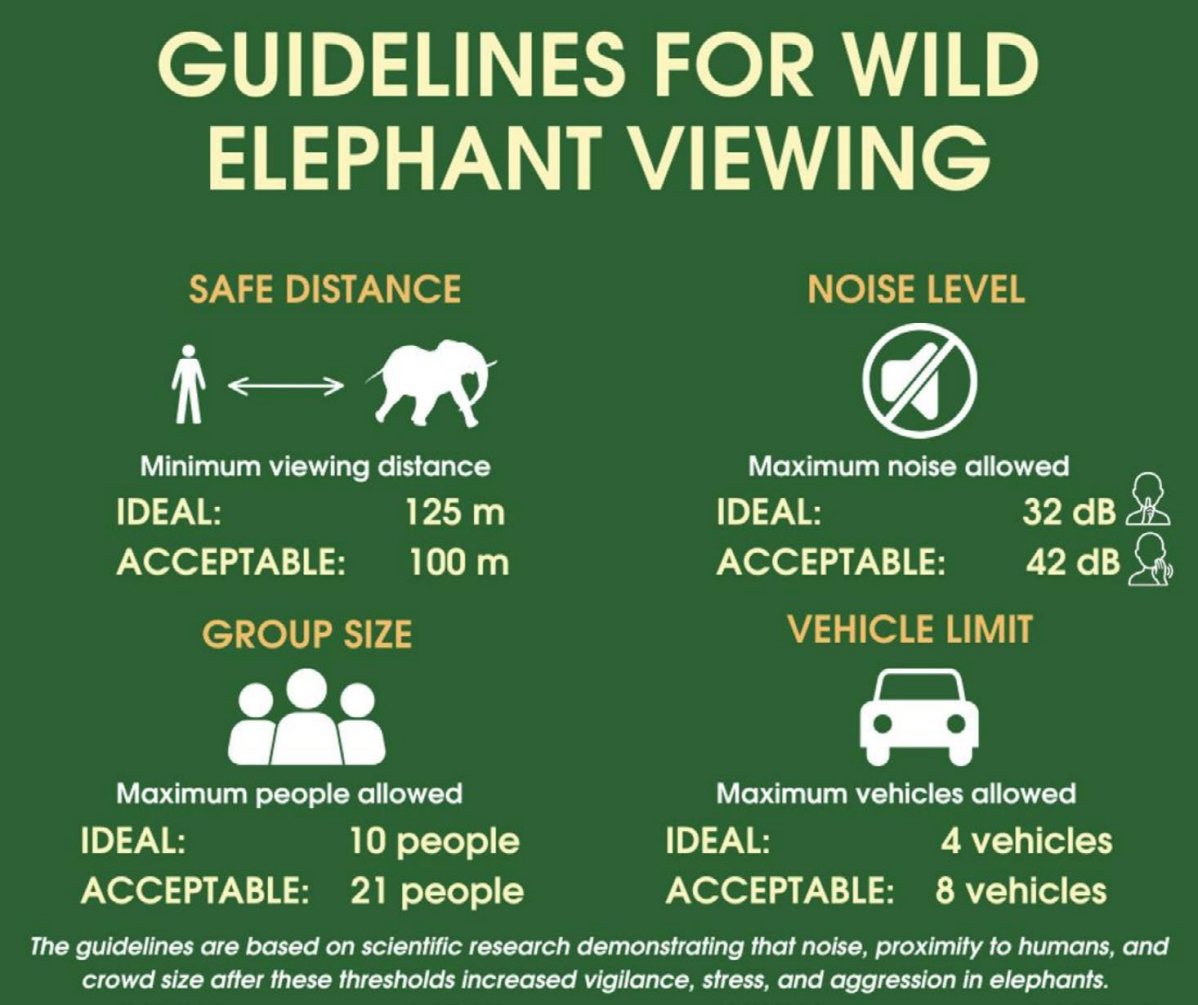
Informational sign outlining evidence‐based guidelines for minimizing behavioral disturbance to elephants during wildlife tourism. These thresholds are derived from empirical research on elephant behavioral responses to anthropogenic disturbance.

Broadly, our findings provide evidence to government entities and protected area managers in establishing science‐based guidelines to ensure sustainable and ethical wildlife viewing (Bansiddhi et al. 2019; Table 6; Figure 7). Thresholds identified in this study offer practical, place‐based regulatory frameworks that contribute to the growing body of literature that emphasizes the importance of regulating human‐wildlife interactions in protected areas (Mumby and Plotnik 2018; Szott, Pretorius, Ganswindt, and Koyama 2019; Tang et al. 2020; Patterson et al. 2025). Outcomes offer critical insights for developing legislation and regulations aiming to balance wildlife protection with the socio‐economic benefits of ecotourism. Guidelines can be developed that increase sustainable and ethical tourism management in KNP but also in similar protected areas with wild elephant viewing in Thailand and other regions of Asia that have wild elephant (or wildlife) tourism, either utilizing these metrics or conducting analyses to determine species and location‐specific thresholds. Integrating these evidence‐based thresholds into park guidelines would enhance elephant welfare and promote more sustainable ecotourism practices (Figure 7). Furthermore, our integration of behavioral observations with regression analyses quantifies the risks associated with various levels of human disturbance, while building on previous methodological insights (Szott, Pretorius, Ganswindt, and Koyama 2019) and could serve as a model for other species and ecosystems with wildlife viewing activities.

Tourist satisfaction in KNP is closely linked to perceptions of environmental quality and wildlife well‐being (Munnab and Sangchoey 2023) with visitors increasingly valuing ethical and sustainable tourism experiences (Higham and Bejder 2008). In 2016, KNP was ranked at an unacceptable level for wildlife tourism management (Khowinthawong and Emphandhu 2016). From the perspective of park personnel, the most important factors included the establishment of laws and legislation to regulate wildlife tourism (Khowinthawong and Emphandhu 2016). Considering this, and to mitigate the impacts of daily and sighting tourism pressure, we recommend implementing the identified thresholds at KNP (Table 6; Figure 7). We also recommend daily limits on visitor and vehicle numbers both within the park and at sightings to maintain a DTP and STP Level of “Medium” and below as defined in this study (Table 2). Buffer zones should be established at the 100/125 m threshold and around ecologically sensitive habitats (Figure 1). Designated quiet viewing areas with appropriate signage and enforcement by staff should be implemented to reduce acoustic disturbance to 32 dB or below (Table 6).

Additional strategies include comprehensive training for guides on recognizing behavioral indicators of stress and aggression in elephants, long‐term monitoring of tourism intensity and behavioral responses, educational awareness for tourists on appropriate behavior, and the impacts of disturbance through signage and engagement. Finally, we recommend adaptive seasonal management such as scheduled park closures during periods of heightened elephant sensitivity including phased and gradual opening/closure protocols to reduce chances of a habituation reset effect. Additionally, a tiered system using behavioral indicators: maintaining DTP/STP ≤ Level 1 or 2 (Table 2) to preserve affiliative interactions in sensitive herd types is recommended.

Overall our findings demonstrate that elephant behavior in KNP is impacted by tourism pressure with clear thresholds in behavior change, consistent with other studies, and, if poorly managed, has the ability to disrupt social interactions and reduce wellbeing (Poole and Moss 2008; Plotnik and De Waal 2014; Pardo et al. 2024; Bates et al. 2025). Considering that even minimal human activity can alter wildlife behavior patterns (Ngoprasert et al. 2017; Benson et al. 2021) and subtle but persistent stress responses may have downstream consequences for energy expenditure, social cohesion, reproductive success, and fitness over time (Szott, Pretorius, and Koyama 2019; Szott 2020; Chock et al. 2025)—it is vital to develop wildlife tourism regulations to prevent these unwanted effects.

Currently, Thailand and other Southeast Asian countries with wild elephant populations lack national‐level legislation specifically regulating wild elephant tourism. In the absence of standardized policies, management practices vary widely across protected areas, potentially leading to inconsistent outcomes for elephant welfare, conservation outcomes, and visitor safety. The behavioral thresholds identified here offer concrete, evidence‐based criteria that could be incorporated into national tourism policies, protected area regulations, or Ministry of Natural Resources and Environment directives by embedding such thresholds into permitting processes, training programs, and enforcement protocols. Additionally, it is recommended that other regions in Thailand, Asia, and beyond, adopt similar baseline thresholds or conduct testing to establish thresholds to protect wildlife in protected areas with wildlife tourism. Adoption of these standards would enhance elephant welfare, visitor safety, and position Thailand as a leader in ethical wildlife tourism. Moreover, these principles could serve as a model for other countries in using wildlife behavioral thresholds to develop guidelines that balance tourism growth and development with wildlife protection and conservation goals.

## Author Contributions

**Brooke Friswold:** conceptualization (equal), data curation (lead), formal analysis (lead), funding acquisition (equal), investigation (lead), methodology (equal), project administration (lead), resources (equal), software (equal), validation (equal), visualization (lead), writing – original draft (lead). **Antoinette van de Water:** conceptualization (equal), data curation (supporting), formal analysis (supporting), investigation (supporting), methodology (supporting), project administration (supporting), resources (supporting), validation (supporting), writing – review and editing (supporting). **Ave Owen:** conceptualization (supporting), data curation (supporting), methodology (supporting), project administration (supporting), resources (supporting), writing – review and editing (supporting). **Megan English:** formal analysis (supporting), methodology (supporting), supervision (supporting), validation (supporting), writing – review and editing (supporting). **Tommaso Savini:** formal analysis (supporting), methodology (supporting), supervision (supporting), validation (supporting), writing – review and editing (supporting). **Liv Baker:** formal analysis (supporting), methodology (supporting), supervision (supporting), validation (supporting), writing – review and editing (supporting). **Chution Savini:** supervision (supporting), validation (supporting), writing – review and editing (supporting). **George Gale:** conceptualization (equal), data curation (supporting), formal analysis (supporting), funding acquisition (equal), investigation (supporting), methodology (equal), project administration (supporting), resources (supporting), supervision (lead), validation (supporting), visualization (supporting), writing – original draft (supporting), writing – review and editing (equal).

## Funding

This work was supported by the King Mongkut's University of Technology Thonburi, Petchra Pra Jom Klao Scholarship and Bring The Elephant Home.

## Disclosure

Inclusion Statement: This study was conducted in Thailand and actively involved collaboration with local stakeholders and researchers. We worked closely with Kuiburi National Park rangers, chiefs and staff as well as Thai research assistants throughout all stages of the project, including study design, data collection, and interpretation of results. Our author team includes contributors from both Thailand and other countries, reflecting a diverse set of perspectives. Intellectual input from Thai collaborators was integral to the development and contextualization of the study, and we ensured that relevant literature from the region was cited. We are committed to sharing the results with park management, local staff, and broader conservation stakeholders in Thailand to support evidence‐based wildlife tourism practices.

## Conflicts of Interest

The authors declare no conflicts of interest.

## Supporting information


**Data S1:** ece372842‐sup‐0001‐Supinfo.zip.

## Data Availability

All the required data are uploaded as Supporting Information and have been included in the submission.
