# Supplementary material for: Impacts of Tourism on Wild Elephant Behavior in a Protected Area: Thresholds for Sustainable Wildlife Viewing
Source: Ecol Evol. 2026 Jan 12;16(1):e72842. doi: 10.1002/ece3.72842 (PMC12793899; doi:10.1002/ece3.72842)
Supplement: Supplementary file 1 — Data S1: ece372842‐sup‐0001‐Supinfo.zip. [file ECE3-16-e72842-s001.zip › Supplement.docx]

**Supplementary Materials**

**Table 1:** Ethogram of behaviors and their respective behavior categories and definitions.

| **Continuous Behavior** | | |
| --- | --- | --- |
| **Vigilance** | Alert | Standing with head raised, ears spread, tail raised, trunk raised or turned in a 'sniff' position. Often the head is moved from one side to another such as to listen to their surroundings. |
| **Stress-related** | Flee / Retreat | Rapidly or intentionally moving away from a conspecific/animal/human. Fleeing may also occur when an elephant is startled which includes running in the opposite direction. |
| **Affiliative behavior** | Approach | An elephant directly approaching and attempting to engage with a conspecific, animal, or human. |
|  | Nursing | A calf nursing from a lactating mother. |
|  | Physical interaction | An elephant directly interacts with a conspecific physically. |
|  | Play spar | Head-to-head with raised chins, pulling and pushing with intertwined trunks. Included mouth wrestling with trunks thrown back over the head. Intensity level is less compared to actual sparring. |
|  | Reproductive behavior | Interaction of reproduction behavior between conspecifics (mounting, sparring, mating, continued genital touching). |
| **Passive aggressive** | Follow | Following a conspecific, human/vehicle, or animal from close behind in a slowed or medium pace. Often accompanied by genital touching or trunk over back. |
| **Active aggressive** | Chase | Rapid pursuit of a conspecific, animal, or human/vehicle, which responds by actively moving in a direction away from the initiator. |
|  | Spar | Individuals face each other head-to-head with raised chins, pulling and pushing with intertwined trunks. Includes mouth wrestling with trunks thrown back over the head including other behaviors intended to harm or dominate. |
| **Relaxed** | Food and drink | Behaviors related to consumption including: Drinking, Browsing, Grazing, Rooting, and Digging. |
|  | Locomotion | Movement from one place to another. |
|  | Inactive | Not expressing any particular behavior but in a relaxed state with ears and tail relaxed. |
|  | Self maintenance | Engaging in self cleaning and maintenance behavior including: Bathing, Rubbing, Dusting, Wallowing and Soiling. |
|  | Rest | Lying down or sleeping leaned against something. |
|  | Manipulating object | Playing with or pushing around an object (i.e. tree stump, grass, etc.) in a non-aggressive manner. |
|  | Playing alone | Splashing, rolling, or other self-directed pleasure-seeking behavior. |
| **Other** | Out of sight | Focal animal cannot be observed. |
| **All-occurrence behaviors** | | |
| **Vigilance** | Smell | Animal extends the trunk down or up, with the tip of the trunk curled horizontally toward stimulus. Often the trunk is rotated in several directions to pick up scent. |
|  | Tail stiff and erect | Tail is raised high up in the air or sticking straight out from behind the body. |
|  | Trunk to mouth | Placing the tip of the trunk into its own mouth without ingesting any food or water, possibly processing chemicals using its vomeronasal organ. |
| **Stress-related** | Trunk to body | Touching their own body with a trunk. Different from scratching, gathering food, or cleaning. Minimal of 3 second intervals between two consecutive touch face behaviors. |
|  | Touch face | Touching their own face including: Trunk, Tusk, or Temporal gland with the trunk. Minimal of 3 second intervals between two consecutive touch face behaviors. |
|  | Trunk curl / J trunk / Trunk swinging | Curling trunk not related to consumption, self maintenance, or play. Holding the trunk in a J position often with the head held high and during locomotion. Swinging trunk in between front legs not related to consumption, self maintenance, or play. Minimal of 3 second intervals between two consecutive touch face behaviors. |
|  | Foot swinging / Foot swaying / Foot scuffing | Swinging or swaying foot (often front foot) back and forth or scuffing the dirt in front of them. Not related to digging or foraging. Minimal of 3 second intervals between two consecutive touch face behaviors. |
| **Affiliative** | Head rubbing | Rubbing head, face, or body against a conspecific. |
|  | Mating | Mounting and penetration between a cow and a bull. |
|  | Sweeping | Placing the trunk over the back or head of a conspecific and moving the trunk in a sweeping motion often has been mother and calf. Not to be confused with the aggressive behavior of 'Trunk over back'. |
|  | Trunk intertwine | Head-to-head conspecifics will intertwine trunks. Not to be confused with 'Play sparring' or 'Sparring'. |
|  | Trunk to another's body | Trunk tip touching a particular part of the body of a conspecific. |
|  | Trunk to another's genital | Trunk tip touching the genital area of a conspecific. |
|  | Trunk to another's head | Trunk tip touching a particular part of the face of a conspecific including: Palatal pit, Temporal gland, Mouth etc. |
| **Passive Aggressive** | Displace | Initiator approaches conspecific moving them from their current position. Position of the recipient is then occupied by the initiator within one body length. No physical contact. |
|  | Ear slap/ear fold | Using both ears to slap the sides of their body to make an audible sound. Folding the tops of the ears backwards or curling the bottom of ears upward. Usually directed towards a conspecific, human/vehicle, or wildlife. Not to be confused with general dusting or heat regulation. |
|  | Head shake | Head above shoulders and shaken vigorously from side to side causing the trunk to move vigorously and ears flapping against the body. Can be done whilst turning towards or away from a conspecific, human/vehicle, and animal at which it is directed. |
|  | Head up and ears out | Head held up above shoulders, trunk up and ears out to intimidate conspecific, human/vehicle, or animal. Can occur with a head shake, or in play situations. Distinction: in play, it will be directed at objects. |
|  | Trumpet blast | Loud and sudden blast of trumpet, often in protest, distress, aggression or pursuit. |
|  | Water spray | Expulsion of water in the direction of a conspecific, human/vehicle, or animal. |
| **Active Aggressive** | Bite | Putting the mouth around and applying force to a conspecific. |
|  | Charge | Rapid, forward lunging motion or rapid gait toward a conspecific, human/vehicle, or animal with head held above shoulders, and ears held perpendicular to the body. Can be a 'Mock Charge' where the trunk is up and pursuit ends quickly or a 'Full Charge' where the trunk is curled in and pursuit continues. |
|  | Drive | Placing head or tusks to rear, back or side of a conspecific resulting in a continual displacement of at least one body length. |
|  | Head butt | Butting forehead or base of trunk against body or forehead of a conspecific. |
|  | Kick | Kicking with forefoot or hind foot towards a conspecific, human/vehicle, animal, or inanimate object. |
|  | Push down on head | Pushing down on a conspecific's head with the base of trunk, tusks, or open mouth. |
|  | Trunk hit | Quick sharp contact to a conspecific, human/vehicle, or animal with the dorsal side of the distal end of the trunk. |
|  | Tusk | Pushing or attempting to push tusks into a conspecific's body accompanied by a forward, lunging motion. Can also be directed at the ground particularly during musth. |
|  | Mount | Raising the forelegs and placing them along the back of a conspecific from the rear in a dominance display. Not to be confused with mating behavior. |
|  | Trunk over back | Placing at least two-thirds of the trunk over the back, head, neck, or side of a conspecific with a high degree of tension in the trunk tip.. Not to be confused with affiliative 'Sweeping' behavior. |
|  | Push / throw / kick object | Pushing an object with its body in act of aggression or throwing/kicking an object toward a conspecific, human/vehicle, or animal. Not to be confused with the solitary behavior of 'Playing alone' or 'Manipulating object.' |
|  | Event | An event or stimulus that an elephant(s) respond to (i.e. loud noise, human/vehicle approach, wildlife interaction etc.). |


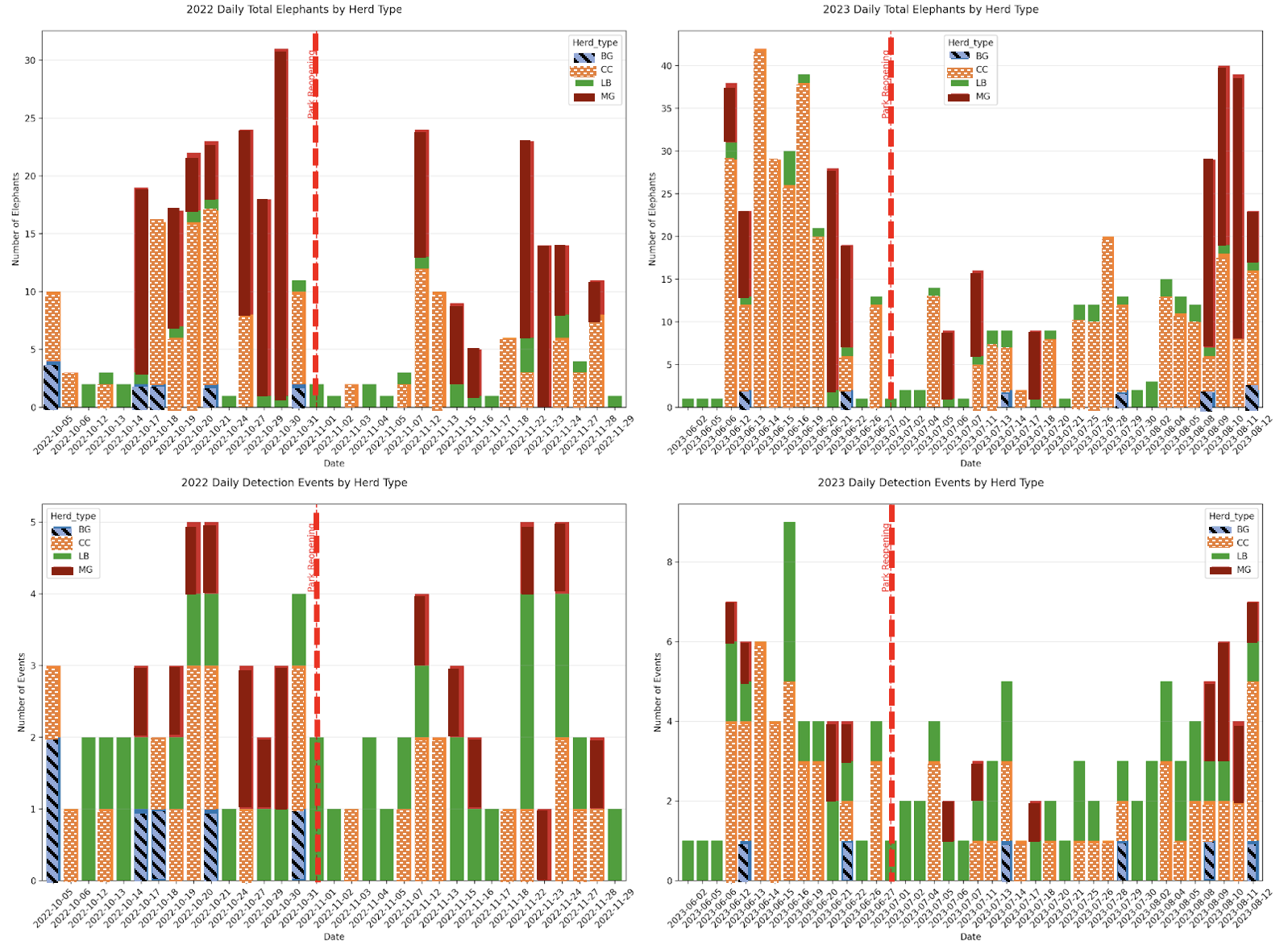


**Figure 1:** Daily elephant detections by herd composition during pre and post park re-opening periods in 2022 and 2023. The stacked bar plots show (**Top Left**) total elephants detected per day in 2022, (**Top Right**) total elephants detected per day in 2023, (**Bottom Left**) number of detection events per day in 2022, and (**Bottom Right**) number of detection events per day in 2023. Herd types are categorized as Bull Group (BG), Cow-Calf Group (CC), Lone Bull (LB), and Mixed Group (MG). Red dashed vertical lines indicate park re-opening dates (November 1, 2022, and July 1, 2023). Each bar represents the daily total, with colors indicating herd types.


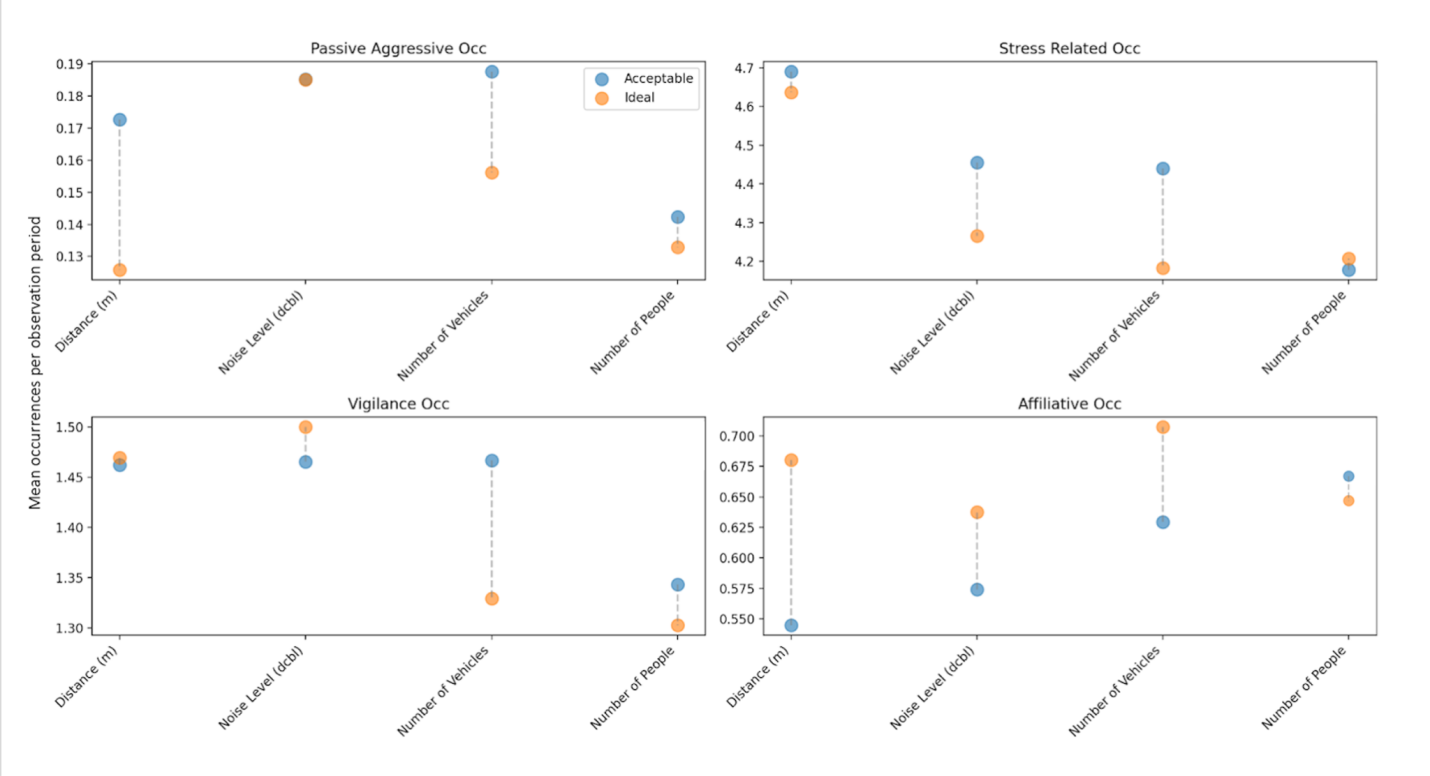


**Figure 2:** Mean behavioral response rates of elephants from identified “ideal” to “acceptable thresholds showing the difference in behavior change between them for four behavioral categories: (**Top left**) Passive Aggressive; (**Top right**) Stress-Related; (**Bottom left**) Vigilance; and (**Bottom right**) Affiliative. Compared against four anthropogenic predictors (x-axis) noise level (dB), distance to people (m), number of vehicles, and number of people present. Dashed lines indicate the magnitude and direction of behavioral shifts associated with the difference between ideal and acceptable conditions.
